# Proteomic analysis of somatic embryo development in *Musa* spp. cv. Grand Naine (AAA)

**DOI:** 10.1038/s41598-020-61005-2

**Published:** 2020-03-11

**Authors:** Marimuthu Kumaravel, Subbaraya Uma, Suthanthiram Backiyarani, Marimuthu Somasundaram Saraswathi

**Affiliations:** 1 Crop Improvement Division, ICAR-National Research Centre for Banana, Thogamalai Main Road, Thayanoor Post, Tiruchirappalli, 102 Tamil Nadu India; 2 Director, ICAR-National Research Centre for Banana, Thogamalai Main Road, Thayanoor Post, Tiruchirappalli, 102 Tamil Nadu India

**Keywords:** Agricultural genetics, Plant embryogenesis

## Abstract

Somatic embryos are comparable to their zygotic counterparts for morphological traits but are derived from somatic cells through various metabolic regulations, collectively referred as somatic embryogenesis (SE). It has been well exploited for germplasm conservation, genetic engineering, mutation breeding, for artificial seed technology and as a tool for mass multiplication. Though somatic embryo development is an important area of interest in growth, and developmental studies, the underlying molecular mechanism remains unclear. Therefore, understanding the molecular basis behind somatic embryo development can provide insight into the signaling pathways integrating this process. Proteomic analysis of somatic embryo development in cv. Grand Naine (AAA) was carried out to identify the differentially expressed protein during somatic embryo development stages, using two dimensional gel electrophoresis together with mass spectrometry. In total, 25 protein spots were differentially expressed during sequential developmental stages of somatic embryos. Among these, three proteins were uniquely present in 30 days globular stage and six proteins in 60 days old mature somatic embryo. Functional annotation of identified spots showed that major proteins are involved in growth and developmental process (17%) followed by defense response (12%) and signal transportation events (12%). In the early stage, cell division and growth related proteins are involved in the induction of somatic embryos whereas in the late developmental stage, cell wall associated proteins along with stress related proteins played a defensive role against dehydration and osmotic stress and resulted in the maturation of somatic embryo. The identified stage specific proteins are valuable indicators and genetic markers for screening and for media manipulation to improve SE efficiency in recalcitrant crops and varieties.

## Introduction

Somatic embryogenesis (SE) is a developmental process, in which somatic cells differentiate into embryos which eventually develop and regenerate into plants. SE is exploited to generate a large quantity of very high economic value, genetically identical and disease free plantlets^1^. SE is considered as the model development system for understanding the various mechanism/s involved in the regulation of plant embryogenesis. The growth and development of somatic embryos are closely related to zygotic embryos by their morphology at different developmental stages like globular, heart shaped, torpedo shaped and cotyledon in dicots and as globular, scutellar and cotyledon/coleoptilar in monocots^2,3^. SE has several advantages over zygotic embryogenesis like ease of monitoring and manipulation of culture strategies for the synchronized development of somatic embryos in large numbers^4^. SE in banana is well reported in many commercial cultivars^5–8^.

Though somatic embryo development is an important area in the growth and development of plant research, clear understanding of the molecular mechanism behind this developmental process still remain elusive. Very little information is known on the sequence of molecular events that enables the conversion of somatic cells into somatic embryos^1,9^. Embryo developmental processes are mainly associated with the morphogenetic events that form the fundamental cellular pattern for the formation of shoot and root primordia and also related to the maturation of embryos through cell growth and storage of reserve energy source^10^. Maturation is the critical stage during somatic embryo development for the conversion of embryo into plantlets as this stage is characterized by cellular differentiation and expansion^9^. However, physiological disorders observed in the emerged embryos and asynchronous developments pose limitation for its wider application. But, understanding the molecular mechanism of somatic embryo development may help in overcoming these limitations and assist commercial exploitation of this phenomenon.

Proteins are mainly responsible for the biological function and phenotype of the cells^10,11^. Proteomic approach has been considered as a powerful tool for examining the physiological condition of plant tissues, and organs, under specific developmental processes^12^. Since proteins are directly involved in cellular biochemistry, identification of proteins related to the embryo development may reveal the molecular basis of SE^13^. Research on the development of somatic embryo has been carried out over three decades, but most of the studies focused on physiological aspects and improvement of culture practice. More recently efforts have been put forth to examine the process of somatic embryo development at the molecular level. Proteomic analysis of different developmental stages of embryos were carried out in many commercial crops like *Citrus sinensis* Osbeck^13^, *Coffea arabica*^1,14^, *Oryza sativa*^10^, *Manihot esculenta*^15^, *Picea glauca*^16^, *Carica papaya* L.^9^, *Cyclamen persicum*^17^, *Araucaria angustifolia*^18^, *Fraxinus mandshurica*^2^ and *Acca sellowiana*^4^. Most of the study revealed that the formation and development of somatic embryos follow complex metabolic process and involve many proteins that are associated with the growth and developmental pathway.

Understanding the molecular basis of somatic embryo development along with signaling pathways can provide insight into the growth and developmental process. Therefore, the study on proteomic analysis of somatic embryo development in *Musa* spp. cv. Grand Naine (AAA) was carried out with the main objective to examine and characterize the differential proteins expressed during various developmental stages of somatic embryo. The determination of differential protein expression will provide a novel insight into banana somatic embryo development and helps in the improvement of SE protocols in recalcitrant banana varieties.

## Materials and Methods

### Plant materials and sample collection

The embryogenic cell suspension (cell line accession no: NGFB0189) generated from male flower bud of cv. Grand Naine (AAA) as per the procedure described by Kumaravel *et al*.^7^ was used as an initiating material. After checking the viability of six months old embryogenic cell suspension using fluroscein diacetate (FDA) stain, one mL settled cell volume (SCV) of fine yellowish white homogenous suspension was plated on the somatic embryo regeneration medium. This was supplemented with 80 µg/L kinetin, 200 µg/L napthaleneacetic acid and 40 µg/L zeatin, as suggested in INIBAP technical guidelines^19^ and maintained in complete darkness at 25 ± 2 °C for 60 days. Five plates of somatic embryos were used as replication. Embryos were collected in triplicates at 0, 30, 45 and 60 days after initiation of embryogenic cells on the regeneration medium. Samples collected were weighed, frozen with LN_2_ and stored in deep freezer for proteomic analysis.

### Protein extraction and 2DE

Proteins were extracted from three biological replicates of each 200 mg of sample as per the phenol ammonium acetate method as described by Kumaravel *et al*.^11^. The extracted protein pellets were resuspended in lysis buffer [7 M Urea, 2 M Thiourea, 4% CHAPS (3-[(3-Cholamidopropyl) dimethylammonio] propanesulfonic acid), 40 mM DTT and 2% IPG buffer (pH 4–7)]. The protein mixture was vortexed for 1 hr at room temperature. The samples were then quantified using 2D quantification kit (GE Healthcare Bio- Science Crop, USA) at 480 nm using Bovine serum albumin (BSA) as standard and purified using 2D clean up kit (GE Healthcare Bio- Science Crop, USA) before applying on immobiline pH gradient (IPG) strips. A total of 200 µg of each sample protein extracts were loaded on to 13 cm IPG strips (pH 4–7) (GE Healthcare Bio-Sciences AB, Sweden) for overnight rehydration. After 12–14 h, the rehydrated strips were subjected to first dimensional separation of proteins using Iso electric focusing (IEF) 100 unit (Hoefer Inc, San Francisco, USA) adopting the IEF conditions described by Kumaravel *et al*.^11^. The focused strips were then subjected to two equilibration process as described by Sharifi *et al*.^3^. Then the equilibrated strips were placed on top of SDS - polyacrylamide gel (12%) for second dimensional separation of proteins using SE 600 unit (Hoefer Inc, San Francisco, USA) at 80 V for 30 min followed by 150 V for 5 h. Three biological replicates were performed for all the samples.

### Staining and image analysis

The gels were stained overnight with colloidal Coomassie Brilliant Blue (CBB) stain followed by destaining with several washes of distilled water. The destained gels were documented using EPSON scanner (EPSON PERFECTION V 750 PRO, Seiko Epson Corp, Japan) at 800 dpi. The documented images were analyzed using Hoefer- 2D software (Hoefer Inc, San Francisco, USA) and spots with more than 1.5 folds were selected for further analysis. The volumes of reproducible spots from replicate gels were normalized against total spot volume.

### In gel digestion and protein identification

The excised gel spots were sent to Molecular Biophysics Unit, Indian Institute of Science, Bengaluru, India for in-gel digestion and processed for MALDI TOF-TOF (Matrix assisted laser desorption ionization time of flight) instrument (UltrafleXtreme, Bruker Daltonics, Germany). The steps were followed based on the instructions available on the website (http://proteomics.mbu.iisc.ac.in/). The PMF and MS/MS data were analysed using Flex analysis 3.1 software. The proteins were identified using MASCOT tool (www.matrixscience.com) and the search parameters were set as suggested by Kumaravel *et al*.^11^. Finally the MS/MS analysis was carried and proteins were identified by performing the following search criteria: Swissprot/ NCBIprot for database, Trypsin for enzyme, no. 1 for missed cleavage, viridiplantae for taxonomy, carbamidomethyl (C) for fixed modification, oxidation (M) for variable modification, 5 to 200 ppm for peptide tolerance, + 1 for peptide charge, default for data format, error tolerant, decoy and precursor, MALDI-TOF-TOF for instrument and auto for report top. The homologous protein spots were again subjected to blast with Banana Genome Hub- South Green (https://banana-genome-hub.southgreen.fr/) to get protein identity. The identified proteins were successfully annotated through BLAST2GO and Uniprot analysis and functionally categorized based on their specific role during embryo development process. The generated protein data has been submitted to the ProteomeXchange Consortium via the proteomics identifications partner repository with the dataset identifier PXD015551^20^.

### RNA isolation, c-DNA synthesis and qRT- PCR analysis

The transcription expression of three genes were analysed by qRT- PCR analysis with Light Cycler 480 instrument (Roche Diagnostics, Germany) using the 2X SYBR Green Real Master Mix (Roche, Germany). The primers were designed and synthesized based on the mRNA sequences encoded for the identified proteins using NCBI primer designing tool (Table 1). RNA was extracted from all four samples like 0, 30, 45 and 60 day somatic embryos using Spectrum Plant Total RNA kit (Sigma-Aldrich, St. Louis, USA) and the concentration was estimated using Colibri spectrometer (Berthold Detection Systems GmbH, Pforzhelm, Germany) by calculating the absorbance at 260 and 280 nm. Using MRN70 miniprep kit (Sigma-Aldrich, St. Louis, USA), mRNA was extracted from total RNA according to manufacturer’s instruction. The first strand cDNA was successfully synthesized from mRNA using cDNA reverse transcription kit (Applied Biosystems, California, USA) with oligo- dT primers. The synthesized cDNA’s were further diluted three fold with double distilled water for qRT- PCR analysis. For each cDNA, 20 µl of reaction volume was set with 5 µM of both forward and reverse primers, 10 µl of 2X master mix and the final volume was made up with nuclease free double distilled water. The endogenous reference gene (RPS2) was used as an internal standard. Thermal cycling was performed as follows: 95 °C for 10 min; 94 °C for 10 s, 59–63 °C for 10 s, 72 °C for 20 s. At the end of PCR, the transcriptional expression level of each gene was quantified based on the normalized ratio with advanced relative quantitation.

**Table 1:** List of primers used in quantitative RT-PCR.

Protein	Forward Primer	Reverse Primer
Late embryogenesis abundant protein D-34 like (spot 5)	5'AACCGATCGAGATGAGCGAC3'	5'CCTCGTCACGGGTAATTCGT3'
Alpha-amylase isozyme 3D-like (spots 8 and 20)	5'ATTCGTCGACAACCACGACA3'	5'GGAACCCCTGGGTGTGTTAG3'
Pollen coat oleosin-glycine rich protein (spot 18)	5'TGTCGTTGCTGGTGCTATCG3'	5'TCCTTGTACATCCACCACAGC3'

## Results

### Development of somatic embryos

**Figure 1:**
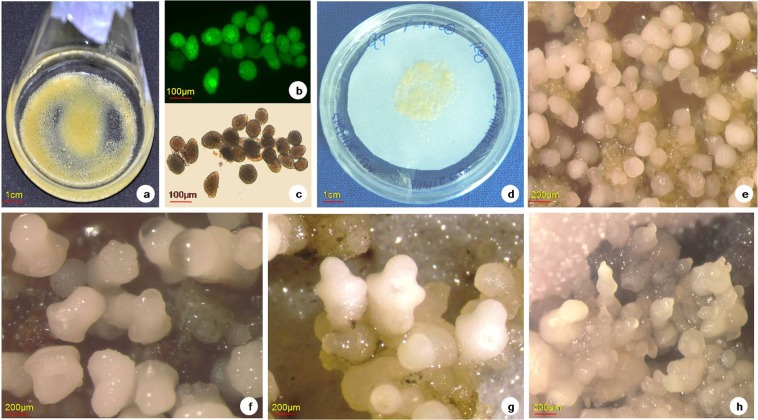
Induction and development of somatic embryo. (**a**) Embryogenic cell suspension (NGFB0189 cell line), (**b**) FDA stained viable cells, (**c**) Unstained normal cells, (**d**) ECS/ 0 day somatic embryo, (**e**) 30 days globular somatic embryo, (**f**) 45 days scutellar somatic embryo, (**g**) 60 days cotyledon stage somatic embryo, (**h**) 60 days coleoptilar stage somatic embryo.

Somatic embryos were induced from the embryogenic cell suspensions that were plated on the regeneration medium supplemented with zeatin and incubated under dark condition. The incubated plates were monitored at regular intervals 30, 45 and 60 days. The formation of globular somatic embryos at 30 days (Fig. 1e), scutellar staged embryo at 45 days (Fig. 1f) and cotyledon/coleoptilar staged embryo at 60 days (Figure g and h) were observed. On an average, around 5877 mature somatic embryos were produced from one mL SCV of NGFB0189 embryogenic cell line.

### Proteomic analysis

**Figure 2:**
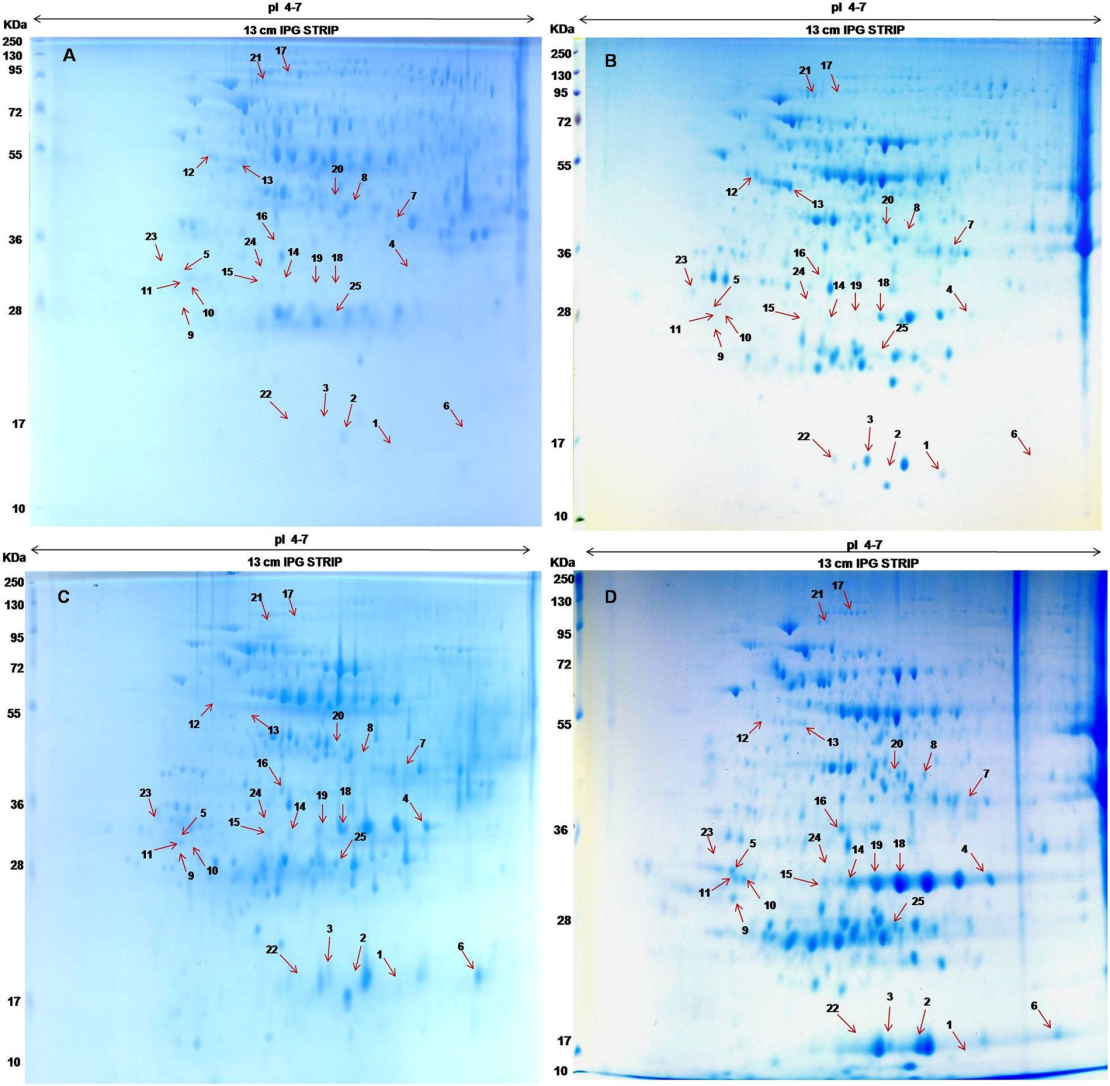
Two Dimensional gel images. (**a**) 0^th^ Day somatic embryo, (**b**) 30^th^ Day somatic embryo, (**c**) 45^th^ Day somatic embryo and (**d**) 60^th^ Day somatic embryo.

Total proteins were extracted from different developmental stages viz., ECS/0, 30, 45 and 60 day somatic embryo (Dse) samples and analyzed using 2DE. In total 25 spots were differentially accumulated during the developmental stages of somatic embryos (Fig. 2). Gel replicates of all the samples are given in the supplementary data info file I. Among them, three proteins (spots 1, 22 and 24) were uniquely present in 30 days globular staged somatic embryos and six proteins (spots 2, 5, 8, 9, 10 and 11) in 60 days mature somatic embryo. Quantitative analysis showed that five proteins (spots 3, 7, 12, 13 and 21), two proteins (spots 6 and 23) and nine proteins (spots 4, 14, 15, 16, 17, 18, 19, 20 and 25) were highly abundant in 30 days, 45 days and 60 days old somatic embryos, respectively. The statistical data of the analyzed spots are presented in the supplementary data info file II. The theoretical MWs of maximum number of identified spots matched with the experimental MWs except for six spots (1, 4, 17, 21, 22 and 24) which showed variations in experimental MWs due to possible protein degradation. Low correlation was also found between the theoretical and experimental pI values of three identified protein spots (1, 18 and 23). The reasons could be that the experimental values were directly analysed from the gel images that are allowed for perturbation and possible for variation in pH gradient across rehydrated gel strips and difference in protein movement during IEF program.

**Figure 3:**
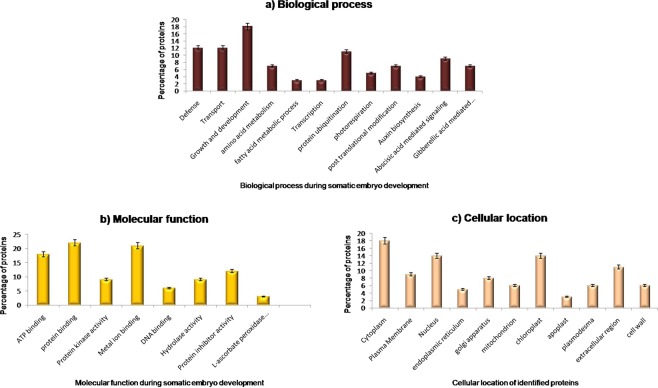
Gene Ontology of differentially accumulated proteins. (**a**) Biological process, (**b**) Molecular function and (**c**) Cellular location.

Among the identified proteins, two forms of pectinesterase (spots 4 and 6), two forms of 14-3-3-like protein GF14-C (spots 10 and11) and two forms of alpha-amylase isozyme 3D-like (spots 8 and 20) were highly accumulated in the mature somatic embryos. The possibilities for correlation of more than one spot to a single protein could be the occurrence of the same protein in different isoforms and/or its post translational modifications^3^. In total, 32% of the identified proteins were found to hit with *Musa* species. The functional classification of identified proteins were carried out by following BLAST2GO and Uniprot analysis which indicated that maximum number of proteins being involved in growth and developmental processes (17%) followed by defense response (12%) and signal transportation events (12%) (Fig. 3). With respect to their molecular functions, around 19% of the proteins were found to be involved in both protein and metal binding activities. Most of the identified proteins were located in cytoplasm (18%), followed by chloroplast and nucleus region (14% each). The list of identified proteins which were differentially accumulated during different stages of somatic embryo development of cv. Grand Naine are represented in Table 2.

**Table 2:** List of identified differentially expressed proteins in developmental stages of somatic embryo in cv. Grand Naine (AAA).

Spot No	Protein Name	Protein view form MASCOT	Protein Id from Banana Genome Hub	Database	Mascot score	Sequence coverage	Taxonomy	Theoretical pI/MW	Experimental pI/MW	Spots pattern (Avg. Normalize volume)
1	Ankyrin protein kinase	gi|224141125	Ma08_p0831 0.1	NCBInr	74	31%	*Populus trichocarpa*	9.03/53.73	6.2/15	
2	18.1 kDa class I heat shock protein-like	XP_009409760.1	Ma01_p14490.1	NCBIprot	85	48%	*Musa acuminata subsp. malaccensis*	7.71/ 25.71	5.9/17	
3	Pathogenesis-related protein 1-like	XP_009414461.1	Ma08_p34150.1	NCBIprot	100	85%	*Musa acuminata subsp. malaccensis*	5.41/17.67	5.7/16.3	
4	Proteinase inhibitor PTI	IP21_SOLTU	Ma05_p18580.1	SwissProt	50	94%	*Solanum tuberosum*	8.19/6.05	6.35/28.5	
5	Late embryogenesis abundant protein D-34-like	XP_009396738.1	Ma04_p14790.1	NCBIprot	105	59%	*Musa acuminata subsp. malaccensis*	4.78/29.02	4.75/30	
6	Pectinesterase PPME1	PPME1_ARATH	Ma05_p24470.1	SwissProt	40	19%	*Arabidopsis thaliana*	8.74/39.4	6.7/17	
7	Abscisic acid receptor PYL9-like	XP_009603677.1	Ma04_p08530.1	NCBIprot	69	34%	*Nicotiana tomentosiformis*	6.5/21.55	6.25/36	
8	Alpha-amylase isozyme 3D-like	XP_009410104.1	Ma07_p20300.1	NCBIprot	79	29%	*Musa acuminata subsp. malaccensis*	5.63/47.38	6/41	
9	Pectinesterase PPME1	PPME1_ARATH	Ma05_p24470.1	SwissProt	60	31%	*Arabidopsis thaliana*	8.74/39.4	4.8/27	
10	14-3-3-like protein GF14-C	XP_009419276.1	Ma09_p29100.2	NCBIprot	155	64%	*Musa acuminata subsp. malaccensis*	4.79/29.56	4.85/28	
11	14-3-3-like protein GF14-C	XP_020586064.1	Ma09_p29100.2	NCBIprot	86	50%	*Phalaenopsis equestris*	4.79/29.56	4.75/27.5	
12	Growth regulating factor 4	GRF4_ORYSJ	Ma02_p06600.1	SwissProt	40	33%	*Oryza sativa subsp. japonica*	8.58/42.03	5/51.5	
13	Tubulin alpha chain	TBA_PRUDU	Ma06_p02330.1	SwissProt	46	49%	*Prunus dulcis*	4.92/50.18	5.3/48	
14	Fimbrin-2	FIMB2_ARATH	Ma05_p27650.1	SwissProt	62	26%	*Arabidopsis thaliana*	8.52/74.07	5.5/28	
15	Aspartyl aminopeptidase	DNPEP_RICCO	Ma06_p13900.1	SwissProt	59	24%	*Ricinus communis*	6.36/54.32	5.3/28	
16	Ribulose bisphosphate carboxylase small chain 1, chloroplastic	RBS1_SOLTU	Ma02_p24600.1	SwissProt	67	60%	*Solanum tuberosum*	8.23/20.82	5.45/33	
17	Serine/threonine-protein kinase	XP_010530874.1	Ma04_p34740.1	NCBIprot	57	28%	*Tarenaya hassleriana*	4.88/41.65	5.55/103	
18	Pollen coat oleosin-glycine rich protein	AAR15494.1	Ma05_p16840.1	NCBIprot	72	44%	*Arabidopsis arenosa*	10.25/49.58	5.8/28	
19	Patatin-like protein 1	PLP1_ORYSI	Ma10_p25600.1	SwissProt	53	37%	*Oryza sativa subsp. indica*	8.8/44.99	5.65/29	
20	Alpha-amylase isozyme 3D-like	XP_009410104.1	Ma07_p20300.1	NCBIprot	74	35%	*Musa acuminata subsp. malaccensis*	5.63/47.38	5.85/40	
21	Calcium-dependent protein kinase 7-like	XP_003572469.2	Ma03_p22480.1	NCBIprot	68	50%	*Brachypodium distachyon*	6.5/64.2	5.4/95	
22	Pentatricopeptide repeat-containing protein	PP380_ARATH	Ma09_p29860.1	SwissProt	70	23%	*Arabidopsis thaliana*	6.22/110.22	5.5/16	
23	Iron-sulfur protein NUBPL-like isoform X1	XP_013672706.1	Ma07_p18050.2	NCBIprot	78	42%	*Brassica napus*	9.32/41.23	4.7/32	
24	Replication protein A 70 kDa DNA-binding subunit B	RFA1B_ARATH	Ma04_p14340.1	SwissProt	66	28%	*Arabidopsis thaliana*	6.15/67.7	5.35/30	
25	Ascorbate peroxidase	AIP90104.1	Ma07_p15360.1	NCBIprot	79	55%	*Musa AB Group*	5.41/27.42	5.8/25.25	

**Figure 4:**
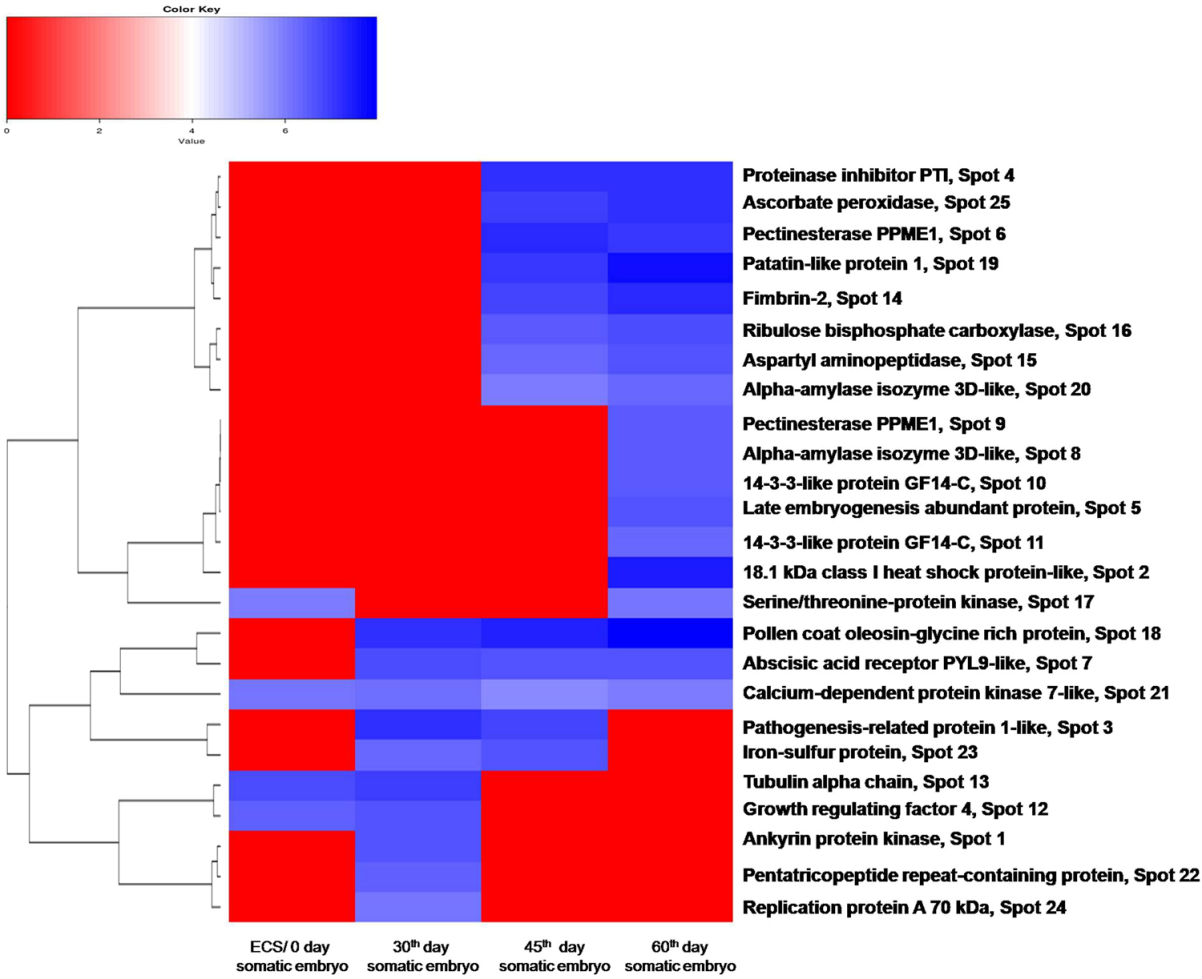
Hierarchical clustering of differentially accumulated proteins. The heat map represents the protein expression based on the level of average normalized volume of spots.

The clustering of differentially accumulated proteins were based on their expression levels displayed during the developmental stages. The expression pattern of proteins were based on the intensity of the spot average normalized volume and are expressed using linear scale. The larger number 6 (maximum positive expression: blue) represents the higher expression and the least value 0 (maximum positive expression: red) representing lower expression. Around 60% of the identified proteins were highly expressed in mature somatic embryos, indicated in the blue color of the map (Fig. 4).

**Figure 5:**
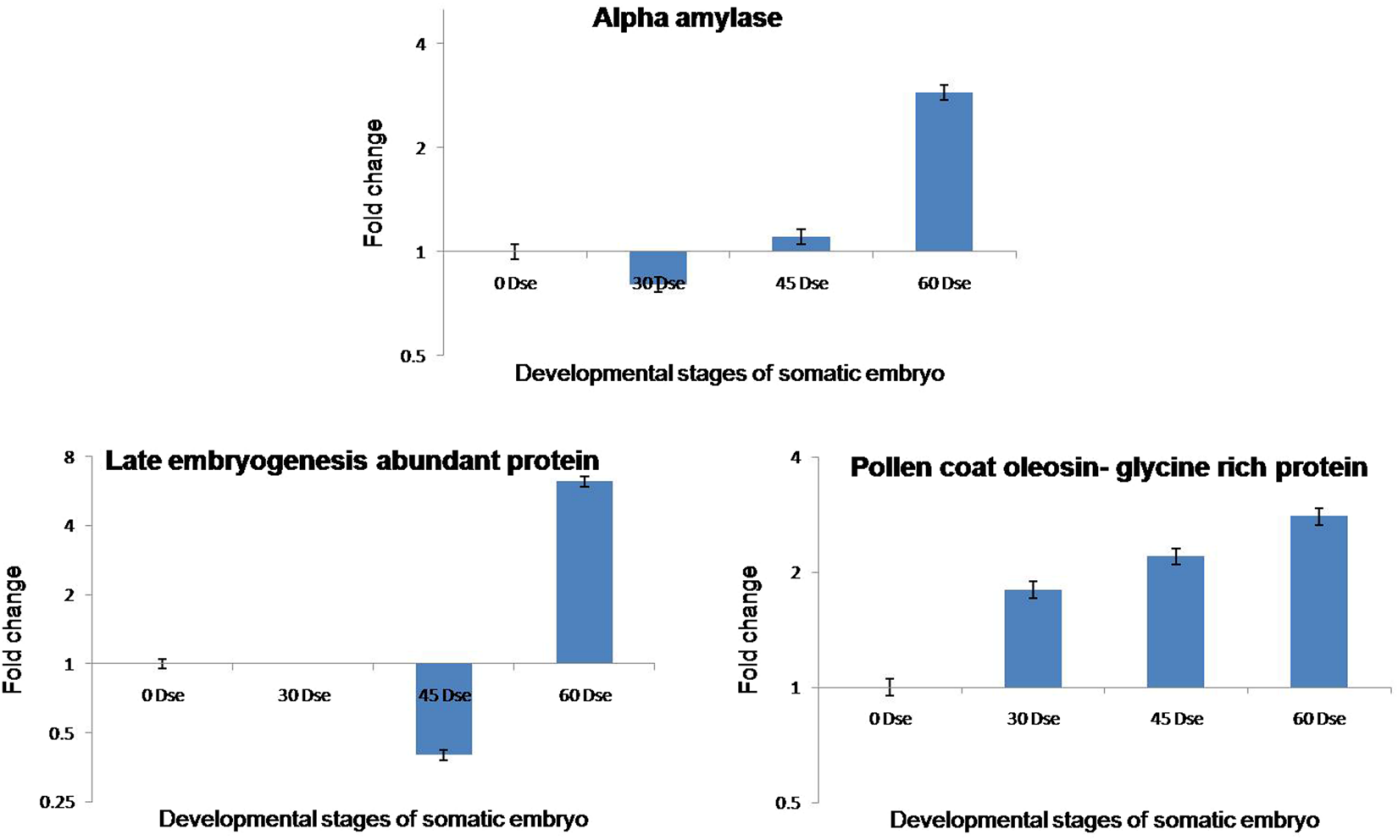
Quantitative RT-PCR validation of 2DE protein results. Relative quantification was carried out to measure fold changes in selected gene expression among 0, 30, 45 and 60 Dse relative to internal reference gene.RPS2 was used as a reference gene. Data (technical replicates of three biological experiments) are reported as means ± standard error.

The validation of proteins identified with respect to the transcripts was carried out for three differentially accumulated proteins- alpha amylase isozyme 3D-like protein (spots 8 and 20), late embryogenesis abundant protein (LEA, spot 5) and pollen coat oleosin glycine rich protein (spot 18) are highly expressed in mature somatic embryos, were selected for quantitative real time PCR analysis. Ribosomal protein (RPS2) gene was used as the internal standard throughout the experiment as it has been validated as the most suitable reference gene in banana^21^. The transcript expression level of LEA and pollen coat oleosin glycine rich proteins showed good correlation with the protein expression found in the 2D gels. In mature somatic embryo, LEA recorded a maximum of 6.2 folds changes in expression and pollen coat oleosin glycine rich protein showed 2.8 folds change in expression (Fig. 5). Though the transcript level of alpha amylase correlated well with the protein expression in mature embryo, we failed to record a correlation between protein expression at transcript level, during the globular stage of the somatic embryo development. This lack of correlation is attributed to the possible post translational modification of proteins and unstable nature of mRNA^22,23^.

## Discussion

Somatic embryo development is a complex process in which many proteins are associated with induction, development and maturation. In the present study, we have identified 25 proteins that are differentially expressed between the developmental stages of somatic embryo of cv. Grand Naine. The function of the identified proteins during specific stage of development has been briefly discussed below.

### Proteins involved in early stages of somatic embryo development

Two cytoskeleton related proteins, like ankyrin protein kinase (spot 1) and tubulin alpha chain (spot 13), were highly expressed during the globular stage (30 days) of somatic embryo development. Ankyrin protein kinase is adaptor proteins that associate the membrane proteins to the cytoskeleton proteins, expressed only in the globular stage somatic embryos. These ankyrin proteins form a complex network consisting of signaling molecules, membrane proteins and cytoskeletal components resulting in the establishment of infrastructure of membrane domains with specific functions^24^. The membrane cytoskeleton is reported to be a key factor for maintaining the shape of cells and avoiding lateral dispersion of integral membrane proteins^25^. Ankyrins are involved mainly in the attachment of cytoskeleton to the plasma membrane of the cells of developing somatic embryos through vertical interactions, while, cytoskeletons are known to be actively involved in embryo polarization and formation. The linkage between plasma membrane, cytoskeleton, cell wall continuum and other associated adhesive domain playing a crucial role in the regulation of early somatic embryo formation^26^. Tubulin alpha chain (spot 13) was found to be upregulated in globular stage embryo. The alpha tubulin protein, one of the building blocks of microtubules, is known to play a major role in cell division and mobility, intracellular transport and the regulation of cell shape^27^. The expression of alpha tubulin was observed during the somatic embryo development of *Manihot esculenta* Crantz^15^ and played a role in creating cellular division planes. In contrast, the expression level of alpha tubulin was highly abundant in later stage than the early stages of somatic embryo development in carrot and found that the level of tubulin proteins increasing simultaneously with the increase in cell size during the developmental phase^28^. Hence it is speculated that ankyrin protein kinase along with tubulin alpha chain plays a major role in the association of cytoskeleton with the plasma membrane and microtubule formation of newly divided cells during the early somatic embryo development of banana.

Pathogenesis related (PR) protein (spot 3) was found to be overexpressed in the globular stage somatic embryo. Apart from being involved in the plant defense system, PR proteins are known to be associated with organ and tissue developmental program^29^. Often PR proteins are highly expressed during osmotic stress conditions. In accordance with our results, PR proteins are reported to be highly expressed during the somatic embryo development of various species like *Vitis vinifera*^30^, *Vitis pseudoreticulata*^31^, *Cichorium*^29^ and *Pinus nigra* Arn.^32^. In *Cichorium*, callose present in the cell wall of embryogenic cells was degraded and disappeared by the involvement of PR proteins and played a significant role in embryo development^29^. Therefore, we present that PR proteins together with cytoskeleton related proteins are necessary for the stabilization of cytoskeleton and modification of cell wall during initial stages of banana somatic embryo development.

Growth regulating factor 4 (spot 12) was also highly expressed at globular stage of the embryo which plays an important role in controlling growth under various stress conditions and known for regulating the plant longevity. In *Arabidopsis*, atgrf4 which belongs to small transcription family, involved in both cell proliferation as well as embryonic development^33^. Growth factor plays a vital role in controlling cell fate and performance during the developmental process^34^. Calcium-dependent protein kinase 7-like (spot 21) protein involved in calcium ion signaling pathway was found to be overexpressed in globular embryos. CDPK are encoded by multigene family and are implicated to participate in the regulatory functions of various developmental and metabolic events. In support to our findings, the expression of CDPK isoform was found to be highly expressed in early developmental process of embryogenesis in sandalwood^35^. Kiselev *et al*.^36^ demonstrated that CDPK was highly expressed during SE of *Panax ginseng* and playing a vital role in the development of somatic embryo. Besides embryo development, CDPK is also known to be involved in transmitting calcium signals associated with stress response and regulation of carbohydrate metabolism^36^.

Two nucleic acid binding proteins like Pentatricopeptide repeat-containing (PPR) proteins (spot 22) and replication protein A 70 kDa (spot 24) were overexpressed in globular stage of the somatic embryo. PPR are RNA binding proteins are involved in various processes like transcription, RNA splicing, editing and translation of RNAs into proteins^37^. These PPR proteins were uniquely expressed in globular somatic embryos and may play a crucial role in the synthesis of new proteins related to growth and development of somatic embryos. This PPR protein also acts as an adaptor molecule by processing interaction between cognate transcripts and their effector molecules. The mutants of mitochondrial and chloroplast PPR have been reported in the development of defective embryo development in Arabidopsis^37,38^ clearly indicating the role of PPR in normal development of embryo and organelle biogenesis. Replication protein A 70 kDa is a DNA-binding protein which is uniquely expressed in globular somatic embryo, is involved in various process of DNA metabolism like replication, repair and recombination. Therefore, the results of the present study reveals that nucleic acid binding proteins play a predominant role during early somatic embryo development in banana.

### Proteins involved in late developmental stages of somatic embryo

During later stages of somatic embryo development, higher accumulation of three stress related proteins, which includes 18.1 kDa class I heat shock protein (spot 2), late embryogenesis abundant (LEA) protein (spot 5) and ascorbate peroxidase (APOX) (spot 25) were observed in mature cotyledon stage of somatic embryo development. Small heat shock proteins (sHSP) are molecular chaperons and engaged in dessication tolerance of somatic embryo. 18.1 kDa heat shock protein is one of the sHSPs which was uniquely expressed in mature cotyledon stage of somatic embryo. These sHSPs are associated with membranes, cytoskeleton and nucleus, mainly involved in protein – protein interactions like folding of partially folded and denatured proteins. They play a key role in proper functional protein conformation and restraining unwanted irreversible protein complexes^39^. These cytosolic sHSPs are highly expressed in mature somatic embryos of various species like alfaalfa, arabidopsis, maize, pea, sunflower, tobacco, tomato and wheat^40^. In *Picea asperata*, 18.1 kDa HSP was upregulated during the partial desiccation of somatic embryo and was related to the elevated levels of hydrogen peroxide and embryo development^41^. Transient accumulation of sHSP has been reported during the maturation of somatic embryo of cork oak^42^. Based on the characteristics of these chaperones, sHSP may be regarded as one of the functional components in the somatic embryo maturation.

Late embryogenesis abundant (LEA) protein was uniquely expressed in 60 days old somatic embryos. Since LEA proteins are highly expressed in mature somatic embryo and are considered to play a pivotal role in late embryonic development. These proteins are also known as late embryogenesis abundant protein^43^. Important role of LEA protein in cellular protection during various stress tolerance responses including dehydration phase of embryo development is well documented^44,45^. Besides protecting the cellular structure, it is also involved in the protection of proteins from various stress induced damages and also acts as a molecular chaperon by folding of denatured proteins. As incase high expression of LEA protein has been observed in the mature embryos of maize, Norway spruce, chickpea and carrot^43,46–48^. During the maturation process, the developed embryo may undergo cellular expansion and increased dry mass to provide energy for germination^49^.

Ascorbate peroxidase (APOX) is an antioxidant enzyme involved in the removal of H_2_O_2,_ was highly expressed in mature embryos, playing a crucial role in detoxification process. It is closely related to endogenous ascorbic acid that controls cellular metabolisms and also acting as a key regulator of cell division^50^. Apart from its role as antioxidants, APOX is known to be associated with various cellular processes like auxin metabolism, response to various environmental stress, crosslinks in cell wall and involved in the maturity and differentiation of plant tissue and organs^51^. Elevated levels of APOX was reported in the torpedo staged embryo of *Juglans regia* L^52^, microspore derived embryo of *Brassica napus*^53^ and initial phase of somatic embryo development of *Picea glauca*^50^. Thus it is speculated that APOX along with LEA and molecular chaperons plays a defense role against various stresses and could be involved in the maturation of banana somatic embryo.

In this study, two forms of alpha amylase isozyme 3D like protein (spots 8 and 20) were found to be overexpressed in the mature cotyledon stage somatic embryo. Alpha amylase plays a major role in hydrolyzing starch into simple sugars and acts as an energy source for the development of roots and shoots during the germination of embryos^54^. The endogenous Gibberellic acid (GA) secreted in the embryo, along with metabolite signals, regulates the expression of amylase^54,55^. In zygotic embryos, the biosynthesized GA transferred from embryo to aleurone layer, where the alpha amylase secreted gets deposited in endosperm to hydrolyse starch into metabolizable sugar. On the other hand, somatic embryos of monocots like banana and bamboo lack aleurone layer and hence the scutellum of the somatic embryos acts as a potential site for starch deposition and amylase accumulation (Kepczynska and Zielinska, 2006). In *Dendrocalamus hamiltonii*, amylase was highly accumulated in mature somatic embryos and indicated that reduced size of scutellum during embryo maturation could be the result of increased level of amylase^55^. Similar to our present result, high amylase activity was observed in mature somatic embryo of *Triticum aestivum* L and *Crocus sativus* L than in the other developmental stages of somatic embryos^56,57^. Hence it is resumed that starch deposited in the mature somatic embryo of banana was hydrolyzed by the increased activity of alpha amylase and as a result, energy source was provided during the germination of somatic embryos. Two forms of pectinesterases (spots 6 and 9) were overexpressed in late developmental stages of somatic embryos. Pectinesterases are pectinolytic enzymes that hydrolyse the glycosidic bond of pectin substance in the cell wall^58^. Plant cells experience cell expansion during a temporal imbalance between hydraulic pressure of vacuole and extensibility of cell wall results in the increase in cell volume^59^. The pectinesterase enzyme might assist in loosening of the cell wall by degrading pectin, which may result in the cell expansion during maturation and eventual development of the somatic embryo.

Serine /threonine- protein kinase (spot 17) was found to be highly expressed in mature somatic embryo. These kinases are receptor proteins mainly involved in various developmental processes like cell proliferation, modification of cell shape and apoptosis. During embryonic developmental process, these proteins are associated with pattern formation and tissue specification^60^. There are several proteins connected with Serine /threonine- protein kinase transmembrane recptors.14-3-3 like protein was one among the mostly associated proteins with the kinase receptor. In the present study, two forms of 14-3-3-like protein GF14-C (spots 10 and 11) was found to be uniquely expressed in mature somatic embryo. 14-3-3- like proteins are phosphoserine/ phosphothreonine binding proteins that regulate many target proteins through phosphorylation and involved in growth and development of cells^61^. 14-3-3 proteins act as an adaptor involved in cell specific serine/threonine phosphorylation dependent signal cascade^60^. Also 14-3-3- like proteins are involved in various process like regulation of carbohydrates and nitrogen metabolism, activation of protein kinase C and induction of calcium dependent exocytosis^62^. 14-3-3- like proteins were found to be highly expressed in the embryogenic cultures of papaya and two forms of these proteins were highly expressed in the proliferating embryos of Oak and play a protective role against stress generated under *in-vitro* conditions during cell reprogramming. This gives a clear indication that carbon source required for the maturation event may be obtained from the metabolic process of carbohydrates regulated by the highly expressed 14-3-3 like protein. Pollen coat oleosin- glycine rich protein (spot 18) belonging to a class of small proteins related with oil body membrane in plants was overexpressed in mature somatic embryo. The expression of oleosin glycine protein was found to be high during the germination of seeds of *Arabidopsis thaliana* and *Carthamus tinctorius* L.^63,64^. Thus, it is speculated that overexpression of oleosin glycine protein aids the somatic embryo during germination process.

## Conclusion

Somatic embryo development is a highly complex process associated with series of molecular events. Our results showed that the differentially expressed proteins during the developmental stages of somatic embryos are closely related with the various cellular processes. In the early developmental stage, cell division and growth- related proteins like ankyrin protein kinase, tubulin alpha chain and growth regulating factor were involved in the induction of somatic embryos, whereas in the late developmental stage, cell wall associated proteins along with stress related proteins like sHSP, LEA and APOX played a defense role against various abiotic stresses resulting in the maturation of somatic embryo. This knowledge will facilitate towards understanding of metabolic network and molecular mechanisms involved in the developing embryo. Furthermore, some identified stage specific proteins are differentially expressed and are valuable indicators. They can be used as genetic markers to identify the specific stage of somatic embryo development. This study not only helps to understand the molecular basis of somatic embryo development but also will facilitate further experiments in the improvement of germplasm conservation, genetic engineering and vegetative propagation. Henceforth, the lists of differentially expressed proteins in this study provide the fundamental information required for studies on growth and differentiation of somatic embryogenesis process in banana. With these findings, the regeneration medium will be manipulated by supplementing additives that triggers the expression of stage specific proteins responsible for the formation of synchronized matured somatic embryos.

## Supplementary information

Dataset 1.

Dataset 2.

## References

[1] Tonietto A (2012). Proteomic analysis of developing somatic embryos of *Coffea arabica*. Plant. Mol. Biol. Rep..

[2] Liu CP, Yang L, Shen HL (2015). Proteomic analysis of immature *Fraxinus mandshurica* cotyledon tissues during somatic embryogenesis: effects of explant browning on somatic embryogenesis. Int. J. Mol. Sci..

[3] Sharifi G, Ebrahimzadeh H, Ghareyazie B, Gharechahi J, Vatankhah E (2012). Identification of differentially accumulated proteins associated with embryogenic and non-embryogenic calli in saffron (Crocus sativus L.). Proteome Sci..

[4] Cangahuala-Inocente GC, Steiner N, Maldonado SB, Guerra MP (2009). Patterns of protein and carbohydrate accumulation during somatic embryogenesis of *Acca sellowiana*. Pesq. agropec. bras. Brasília.

[5] Ganapathi TR, Suprasanna P, Bapat VA, Kulkarni VM, Rao PS (1999). Somatic embryogenesis and plant regeneration from male flower buds in banana. Curr. Sci..

[6] Akbar, A. Somatic embryogenesis through cell suspension culture in Indian commercial cultivars of Banana (*Musa* spp.) Thesis PhD Bharathidasan university, India, 245pp (2011).

[7] Kumaravel M, Uma S, Backiyarani S, Saraswathi MS (2019). Molecular analysis of somatic embryogenesis through proteomic approach and optimization of protocol in recalcitrant *Musa* spp. Physiologia Plant..

[8] Uma, S., Karthic, R., Kumaravel, M., Backiyarani, S. & Saraswathi, M.S. High- throughput technology for mass production of quality planting material in banana. Shaping the future of horticulture. Chapter four, Kruger Brentt publishers, 35–40 (2019).

[9] Vale EM (2014). Comparative proteomic analysis of somatic embryo maturation in *Carica papaya* L. Proteome Sci..

[10] Xu Hong, Zhang Weiping, Gao Yi, Zhao Yong, Guo Lin, Wang Jianbo (2011). Proteomic analysis of embryo development in rice (Oryza sativa). Planta.

[11] Kumaravel M (2016). Differential proteome analysis during early somatic embryogenesis in *Musa* spp. AAA cv. Grand Naine. Plant. Cell Rep..

[12] Rose JKC, Bashir S, Giovannoni JJ, Jahn MM, Saravanan RS (2004). Tackling the plant proteome: practical approaches, hurdles and experimental tools. Plant. J..

[13] Pan Z, Guan R, Zhu S, Deng X (2009). Proteomic analysis of somatic embryogenesis in Valencia sweet orange (*Citrus sinensis* Osbeck). Plant. Cell Rep..

[14] Yuffa AM, Garcia EG, Nieto MS (1993). Comparative study of protein eleetrophoretic patterns during embryogenesis in *Coffea arabica* cv Catimor. Plant. Cell Rep..

[15] Li K (2010). Proteome characterization of cassava (*Manihot esculenta* Crantz) somatic embryos, plantlets and tuberous roots. Proteome Sci..

[16] Lippert D (2005). Proteome analysis of early somatic embryogenesis in *Picea glauca*. Proteom..

[17] Rode, C., Lindhorst, K., Braun, H.P. & Winkelmann, T. From callus to embryo: a proteomic view on the development and maturation of somatic embryos in *Cyclamen persicum*. *Planta*, 10.1007/s00425-011-1554-1(2011). 10.1007/s00425-011-1554-1 22127736

[18] Balbuena TS (2011). Differential proteome analysis of mature and germinated embryos of *Araucaria angustifolia*. Phytochemistry.

[19] Strosse, H., Domergue, R., Panis, B., Escalant, J. V. & Cote, F. Banana plantain embryogenic cell suspensions. INIBAP technical guidelines 8, in Vezina A, Picq C, International Network for the Improvement of Banana and Plantain, Montpellier, France. P.32 (2003).

[20] Vizcaino JA (2016). Update of PRIDE database and its related tools. Nucleic acid. Res..

[21] Chen L (2011). Validation of reference genes for RT-qPCR studies of gene expression in banana fruit under different experimental conditions. Planta.

[22] Zhang J (2009). Stress response proteins’ differential expression in embryogenic and non-embryogenic callus of Vitis vinifera L. cv. Cabernet Sauvignon-A proteomic approach. Plant. Sci..

[23] Sun L (2013). Comparative proteomic analysis of the H99 inbred maize (*Zea mays* L.) line in embryogenic and non-embryogenic callus during somatic embryogenesis. Plant. Cell Tissue Organ. Cult..

[24] Cunha SR, Mohler PJ (2009). Ankyrin protein networks in membrane formation and stabilization. J. Cell. Mol. Med..

[25] Rubtsov AM, Lopina OD (2000). Ankyrins. FEBS Lett..

[26] Samaj J, Bobak M, Blehova A, Pretova A (2005). Importance of cytoskeleton and cellwall in somatic embryogenesis. Plant. Cell Monogr..

[27] Ludwig SR, Oppenheimer DG, Silflow CD, Snustad DP (1987). Characterization of the a-tubulin gene family of *Arabidopsis thaliana*. Proc. Natl. Acad. Sci. USA.

[28] Cyr RJ, Bustos MM, Guiltinan MJ, Fosket DE (1987). Developmental modulation of tubulin protein during somatic embryogenesis in cultured carrot cells. Planta.

[29] Helleboid S (2000). Three major somatic embryogenesis related proteins in *Cichorium* identified as PR proteins. J. Exp. Botany.

[30] Enoki Shinichi, Suzuki Shunji (2016). Pathogenesis-Related Proteins in Grape. Grape and Wine Biotechnology.

[31] Xu TF (2014). A pathogenesis related protein, VpPR-10.1, from *Vitis pseudoreticulata*: An insight of its mode of antifungal activity. PLoS ONE.

[32] Fraterova L, Salaj T, Matusikova I, Salaj J (2013). The role of chitinases and glucanases in somatic embryogenesis of black pine and hybrid firs. Cent. Eur. J. Biol..

[33] Omidbakhshfard MA, Proost S, Fujikura U, Roeber BM (2015). Growth-regulating factors (grfs): a small transcription factor family with important functions in plant biology. Mol. Plant..

[34] Lovicu FJ, McAvoy JW, Iongh RU (2011). Understanding the role of growth factors in embryonic development: insights from the lens. Phil. Trans. R. Soc. B.

[35] Anil VS, Rao KS (2000). Calcium mediated signaling during sandalwood somatic embryogenesis. Role for exogenous calcium as second messenger. Plant. Physiol..

[36] Kiselev KV, Turlenko AV, Zhuravlev YN (2009). CDPK gene expression in somatic embryos of *Panax ginseng* expressing rolC. Plant. Cell Tiss. Organ. Cult..

[37] Manna S (2015). An overview of pentatricopeptide repeat proteins and their applications. Biochim..

[38] Barkan A, Small I (2014). Pentatricopeptide repeat proteins in plants. Annu. Rev. Plant. Biol..

[39] Waters ER, Lee GJ, Vierling E (1996). Evolution, structure and function of the small heat shock proteins in plants. J. Exp. Botany.

[40] Mahmood T, Safdar W, Abbas BH, Naqvi SMS (2010). An overview on the small heat shock proteins. Afr. J. Biotechnol..

[41] Jing D (2017). Proteomic analysis of stress-related proteins and metabolic pathways in *Picea asperata* somatic embryos during partial desiccation. Plant. Biotechnol. J..

[42] Puigderrajols P (2002). Developmentally and stress induced small heat shock proteins in cork oak somatic embryos. J. Exp. Botany.

[43] Vikrant, Janardhanan P (2018). Progress in understanding the regulation and expression of genes during plant somatic embryogenesis: A review. J. Appl. Biol. Biotechnol..

[44] Campalans A, Pages M, Messeguer R (2000). Protein analysis during almond embryo development. identification and characterization of a late embryogenesis abundant protein. Plant. Physiol. Biochem..

[45] Gao J, Lan T (2015). Functional characterization of the late embryogenesis abundant (LEA) protein gene family from *Pinus tabuliformis* (Pinaceae) in *Escherichia coli*. Sci. Rep..

[46] Thomann EB, Sollinger J, White C, Rivin CJ (1992). Accumulation of group 3 late embryogenesis abundant proteins in *zea mays* embryos. Plant. Physiol..

[47] Vestman, D., *et al*. Important processes during differentiation and early development of somatic embryos of Norway spruce as revealed by changes in global gene expression. *BMC Proceedings*, 10.1186/1753-6561-5-S7-P78 (2011).

[48] Mishra S, Sanyal I, Amla DV (2012). Changes in protein pattern during different developmental stages of somatic embryos in chickpea. Biologia Plant..

[49] Amara I (2014). Insights into Late Embryogenesis Abundant (LEA) Proteins in Plants: From Structure to the Functions. Am. J. Plant. Sci..

[50] Stasolla C, Yeung EC (2001). Ascorbic acid metabolism during white spruce somatic embryo maturation and germination. Physiologia Plant..

[51] Bahmankar Moslem, Mortazavian Seyed Mohammad Mahdi, Tohidfar Masoud, Sadat Noori Seyed Ahmad, Izadi Darbandi Ali, Corrado Giandomenico, Rao Rosa (2017). Chemical Compositions, Somatic Embryogenesis, and Somaclonal Variation in Cumin. BioMed Research International.

[52] Jariteh M, Ebrahimzadeh H, Niknam V, Vahdati K, Amiri R (2011). Antioxidant enzymes activities during secondary somatic embryogenesis in Persian walnut (*Juglans regia* L.). Afr. J. Biotechnol..

[53] Khamiss K, Sabouh M, Kanbar A (2011). Evaluating the impact of brassinolide on ascorbate peroxidase activity during Brassica napus embryo development *in vitro*. J. Appl. Sci. Res..

[54] Kaneko M, Itoh H, Tanaka MU, Ashikari M, Matsuoka M (2002). The α- amylase induction in endosperm during rice seed germination is caused by gibberellin synthesized in epithelium. Plant. Physiol..

[55] Godbole S, Sood A, Sharma M, Nagar PK, Ahuja PS (2004). Starch deposition and amylase accumulation during somatic embryogenesis in bamboo (*Dendrocalamus hamiltonii*). J. Plant. Physiol..

[56] Black M, Corbineau F, Grzesik M, Guy P, Come D (1996). Carbohydrate metabolism in the developing and maturing wheat embryo in relation to its desiccation tolerance. J. Exp. Botany.

[57] Devi K, Sharma M, Ahuja PS (2014). Direct somatic embryogenesis with high frequency plantlet regeneration and successive cormlet production in saffron (*Crocus sativus* L.). South. Afr. J. Botany.

[58] Kohli P, Kalia M, Gupta R (2015). Pectin Methylesterases: A Review. J. Bioprocess. Biotechniques..

[59] Sandt VSTV, Suslov D, Verbelen JP, Vissenberg K (2007). Xyloglucan endotransglucosylase activity loosens a plant cell wall. Ann. Botany.

[60] Shah, K. Characterization of th*e Arabidopsis thaliana* somatic embryogenesis receptor-like kinase 1 protein. Thesis, Van Wageningen Universiteity, Netherland,156pp (2001).

[61] Tchorbadjieva Magdalena I. (2015). Advances in Proteomics of Somatic Embryogenesis. Somatic Embryogenesis in Ornamentals and Its Applications.

[62] Comparot S, Lingiah G, Martin T (2003). Function and specificity of 14-3-3 proteins in the regulation of carbohydrate and nitrogen metabolism. J. Exp. Botany.

[63] Miquel M (2014). Specialization of oleosins in oil body dynamics during seed development in *Arabidopsis* Seeds. Plant. Physiol..

[64] Lu Y (2018). Genome-wide identification, expression profiling, and functional validation of oleosin gene family in *Carthamus tinctorius* l. Front. Plant. Sci..

