# Supplementary material for: Proteomic analysis of somatic embryo development in Musa spp. cv. Grand Naine (AAA)
Source: Sci Rep. 2020 Mar 11;10:4501. doi: 10.1038/s41598-020-61005-2 (PMC7066174; doi:10.1038/s41598-020-61005-2)
Supplement: Supplementary file 1 — Dataset 1. [file 41598_2020_61005_MOESM1_ESM.doc]

**Title Page**

**Title of the Manuscript**

**Proteomic analysis of somatic embryo development in *Musa* spp. cv. Grand Naine (AAA)**

**Affiliations and address of the authors**

1. Mr. Marimuthu Kumaravel, MSc

Senior Research Fellow, Crop Improvement Division,

ICAR-National Research Centre for Banana

Thogamalai Main Road, Thayanoor Post, Tiruchirappalli 102,

Tamil Nadu. India

e-mail: [velu_bai@yahoo.co.in](mailto:velu_bai@yahoo.co.in)

1. Dr. Subbaraya Uma*, Ph.D.

Principal Scientist, Crop Improvement Division,

ICAR-National Research Centre for Banana

Thogamalai Main Road, Thayanoor Post, Tiruchirappalli 102,

Tamil Nadu. India

e-mail: [umabinit@yahoo.co.in](mailto:umabinit@yahoo.co.in)

1. Dr. Suthanthiram Backiyarani, Ph.D.

Principal Scientist, Crop Improvement Division,

ICAR-National Research Centre for Banana

Thogamalai Main Road, Thayanoor Post, Tiruchirappalli 102,

Tamil Nadu. India

e-mail: backiyarani@gmail.com

1. Dr. Marimuthu Somasundaram Saraswathi, Ph.D.

Principal Scientist, Crop Improvement Division,

ICAR-National Research Centre for Banana

Thogamalai Main Road, Thayanoor Post, Tiruchirappalli 102,

Tamil Nadu. India

e-mail: [saraswathimse@gmail.com](mailto:saraswathimse@gmail.com)

**Corresponding Author**

*Dr. Subbaraya Uma, Ph.D.

Principal Scientist, Crop Improvement Division,

ICAR-National Research Centre for Banana

Thogamalai Main Road, Thayanoor Post, Tiruchirappalli 102,

Tamil Nadu. India

e-mail: [umabinit@yahoo.co.in](mailto:umabinit@yahoo.co.in)

Telephone no: 0431-2618125, Mobile no: +919442553117

**Supplementary File I**

2D Gel replicates of the samples (ECS/0, 30, 45 and 60 days somatic embryos (dse)) used for differential proteome analysis.

**Fig.1** **Two Dimension** (**2D) Gel replicates of ECS/0^th^ day somatic embryo**


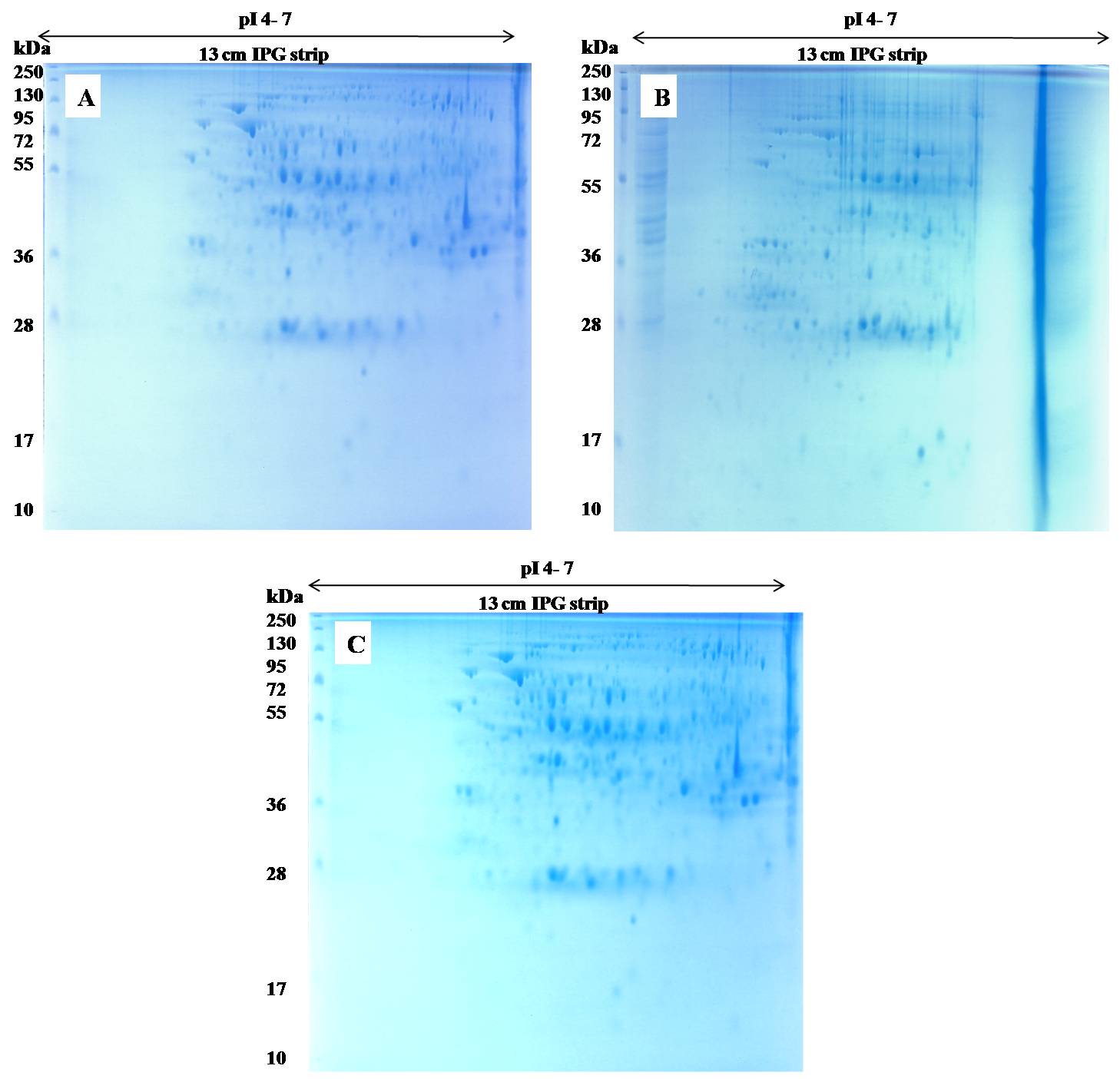


A- First gel replicate of 0^th^ Dse, B- Second gel replicate of 0^th^ Dse and C- Third gel replicate of 0^th^ Dse

**Fig.2** **Two Dimension** (**2D) Gel replicates of 30^th^ day somatic embryo**


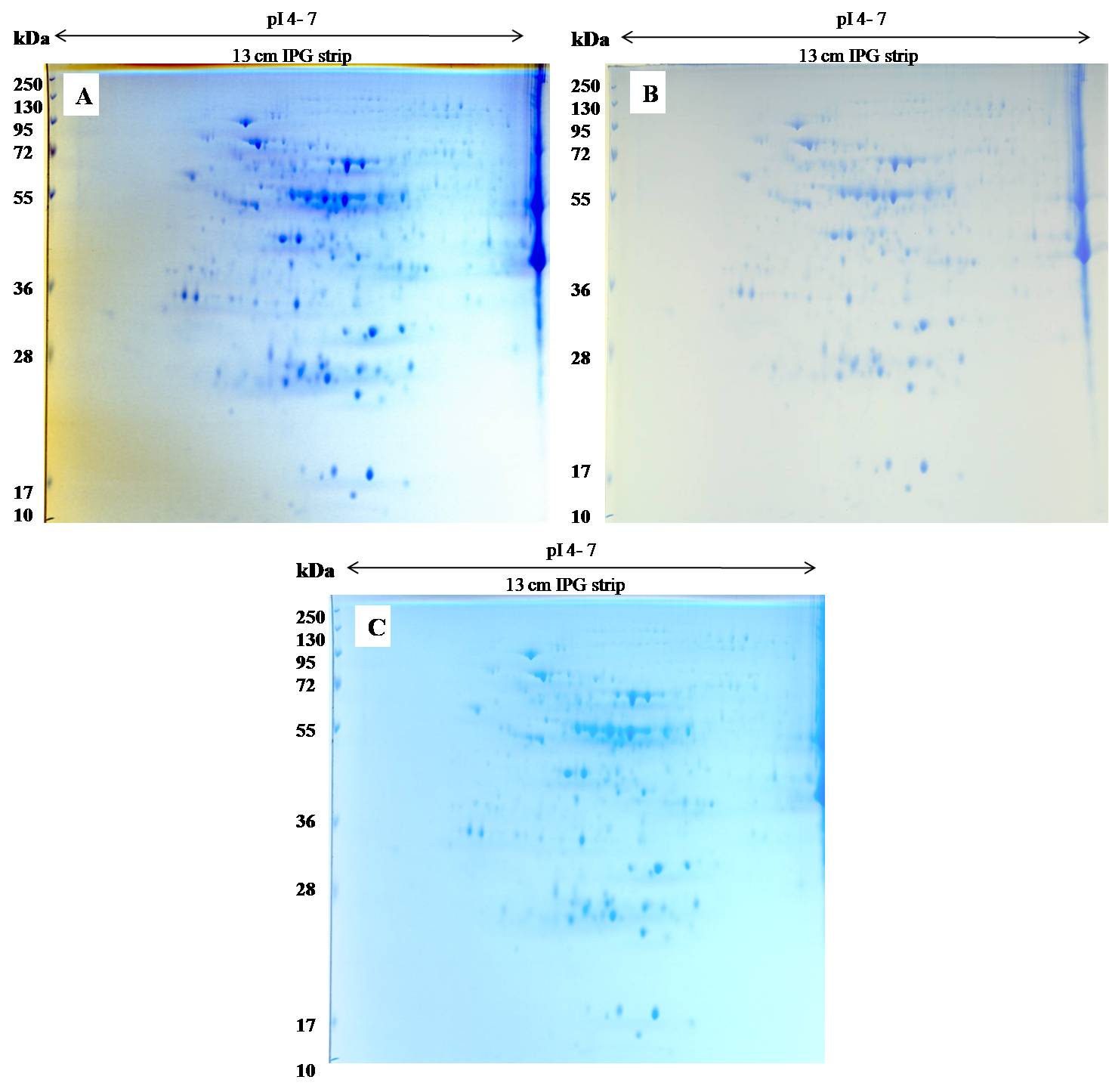


A- First gel replicate of 30^th^ Dse, B- Second gel replicate of 30^th^ Dse and C- Third gel replicate of 30^th^ Dse

**Fig.3** **Two Dimension** (**2D) Gel replicates of 45^th^ day somatic embryo**


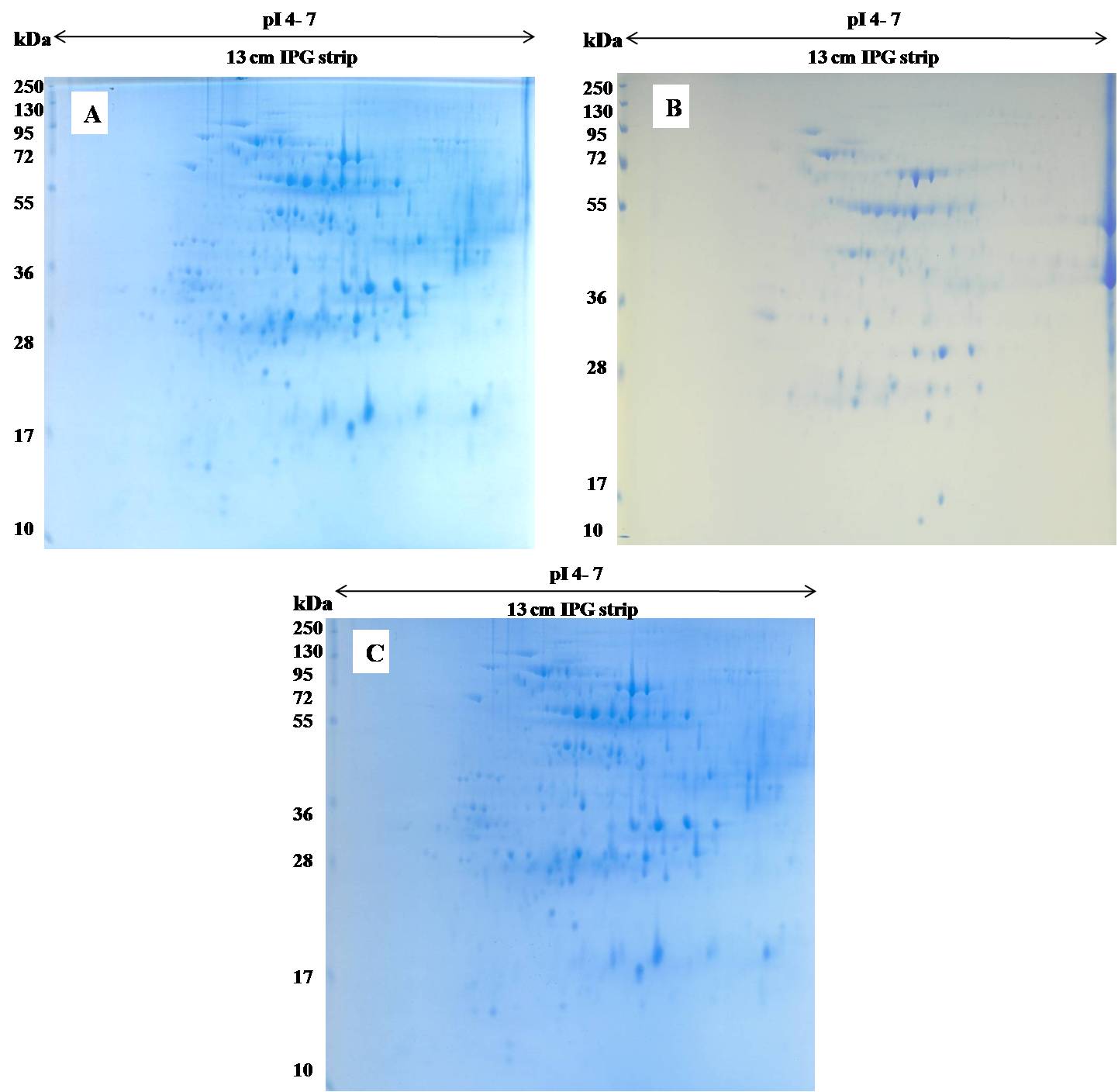


A- First gel replicate of 45^th^ Dse, B- Second gel replicate of 45^th^ Dse and C- Third gel replicate of 45^th^ Dse

**Fig.4** **Two Dimension** (**2D) Gel replicates of 60^th^ day somatic embryo**


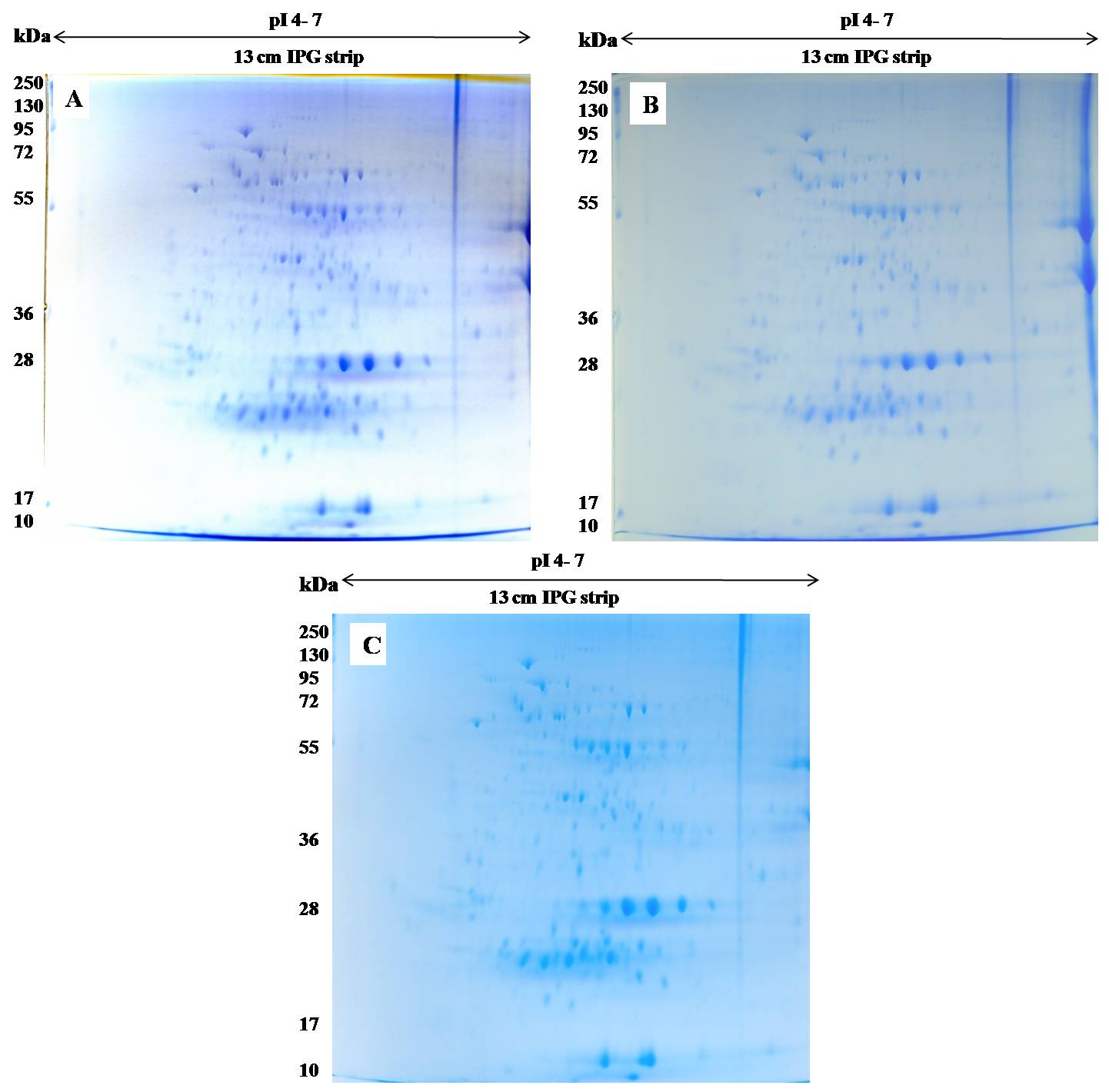


A- First gel replicate of 60^th^ Dse, B- Second gel replicate of 60^th^ Dse and C- Third gel replicate of 60^th^ Dse
