# Supplementary material for: Proteomic analysis of somatic embryo development in Musa spp. cv. Grand Naine (AAA)
Source: Sci Rep. 2020 Mar 11;10:4501. doi: 10.1038/s41598-020-61005-2 (PMC7066174; doi:10.1038/s41598-020-61005-2)

Title Page

Title of the Manuscript

Proteomic analysis of somatic embryo development in *Musa* spp. cv. Grand Naine (AAA)

Affiliations and address of the authors

1) Mr. Marimuthu Kumaravel, MSc

   Senior Research Fellow, Crop Improvement Division,

   ICAR-National Research Centre for Banana

   Thogamalai Main Road, Thayanoor Post, Tiruchirappalli 102,

   Tamil Nadu. India

   e-mail: velu_bai@yahoo.co.in

2) Dr. Subbaraya Uma*, Ph.D.

   Principal Scientist, Crop Improvement Division,

   ICAR-National Research Centre for Banana

   Thogamalai Main Road, Thayanoor Post, Tiruchirappalli 102,

   Tamil Nadu. India

   e-mail: umabinit@yahoo.co.in

3) Dr. Suthanthiram Backiyarani, Ph.D.

   Principal Scientist, Crop Improvement Division,

   ICAR-National Research Centre for Banana

   Thogamalai Main Road, Thayanoor Post, Tiruchirappalli 102,

   Tamil Nadu. India

   e-mail: backiyarani@gmail.com

4) Dr. Marimuthu Somasundaram Saraswathi, Ph.D.

   Principal Scientist, Crop Improvement Division,

   ICAR-National Research Centre for Banana

   Thogamalai Main Road, Thayanoor Post, Tiruchirappalli 102,

   Tamil Nadu. India

   e-mail: saraswathimse@gmail.com

Corresponding Author

Dr. Subbaraya Uma, Ph.D.

Principal Scientist, Crop Improvement Division,

ICAR-National Research Centre for Banana

Thogamalai Main Road, Thayanoor Post, Tiruchirappalli 102,

Tamil Nadu. India

e-mail: umabinit@yahoo.co.in

Telephone no: 0431-2618125, Mobile no: +919442553117

Grand Naine somatic embryo development 2019

Experiment: Grand Naine somatic embryo development 2019

Report created: 6/1/2019 1:32:50 PM

Reference image

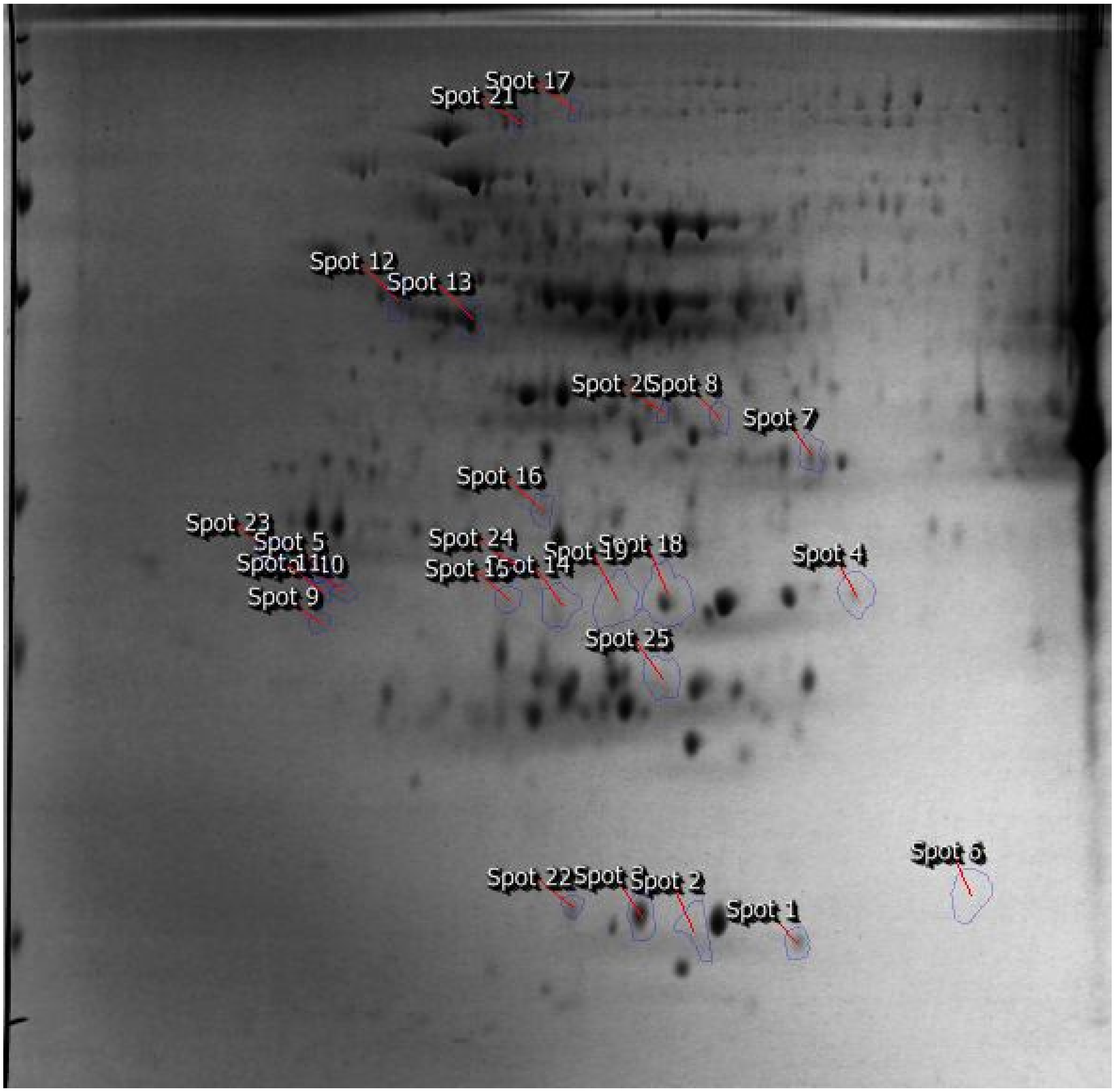

Experiment Design

Group	Grand Naine ECS/0th Day	Grand Naine 30th Day somatic	Grand Naine 45th Day somatic	Grand Naine 60th Day somatic

	somatic embryo	embryos	embryos	embryos
**Replicates**	3	3	3	3

Spots

Anova (p)	Fold	Notes	Average Normalised Volumes			
			Grand Naine ECS/0th Day somatic embryo	Grand Naine 30th Day somatic embryos	Grand Naine 45th Day somatic embryos	Grand Naine 60th Day somatic embryos
7.176e-006	2.1	Spot 17	1.127e+006	6.217e+005	6.160e+005	1.305e+006
4.507e-004	2.4	Spot 21	1.301e+006	1.475e+006	6.243e+005	9.963e+005
3.172e-004	5.6	Spot 20	3.701e+005	5.747e+005	1.065e+006	2.055e+006
0.002	5.5	Spot 23	1.037e+006	2.141e+006	4.035e+006	7.288e+005
5.533e-006	5.8	Spot 4	2.726e+006	5.067e+006	1.394e+007	1.578e+007
4.851e-009	16.5	Spot 18	5.593e+006	1.553e+007	2.341e+007	9.238e+007
4.755e-004	6.1	Spot 19	8.202e+006	7.598e+006	1.275e+007	4.640e+007
5.910e-005	10.1	Spot 9	1.318e+006	1.169e+006	3.807e+005	3.834e+006
1.139e-006	1.8	Spot 25	7.677e+006	1.187e+007	1.011e+007	1.413e+007
3.026e-006	8.1	Spot 6	2.431e+006	3.623e+006	1.966e+007	1.283e+007
1.053e-008	7.7	Spot 3	2.215e+006	1.562e+007	7.498e+006	1.703e+007
9.192e-005	6.1	Spot 1	7.154e+005	4.332e+006	2.363e+006	1.563e+006
1.146e-006	8.2	Spot 2	3.523e+006	6.718e+006	8.062e+006	2.894e+007
2.234e-004	3.6	Spot 5	1.627e+006	1.157e+006	2.189e+006	4.156e+006
9.847e-006	5.5	Spot 7	1.150e+006	6.274e+006	3.862e+006	4.495e+006
0.125	3.0	Spot 8	3.429e+006	2.134e+006	1.257e+006	3.787e+006
3.634e-005	4.4	Spot 10	1.211e+006	1.944e+006	8.522e+005	3.758e+006
1.897e-004	3.7	Spot 11	7.817e+005	5.615e+005	7.522e+005	2.060e+006

3.501e-006	1.9	Spot 12	2.725e+006	3.986e+006	2.063e+006	3.466e+006
7.707e-010	2.5	Spot 13	5.370e+006	8.924e+006	3.612e+006	4.114e+006
1.719e-004	6.3	Spot 14	3.374e+006	7.107e+006	6.537e+006	2.132e+007
5.987e-005	2.5	Spot 15	2.216e+006	1.805e+006	2.205e+006	4.551e+006
1.687e-006	3.4	Spot 16	1.657e+006	2.893e+006	3.772e+006	5.654e+006
2.662e-007	6.0	Spot 22	4.617e+005	2.775e+006	9.992e+005	2.550e+006
0.002	2.1	Spot 24	7.639e+005	1.174e+006	5.675e+005	9.933e+005

Identifier 339

Position (1198, 229)
Notes Spot 17

Grand Naine ECS/0th Day somatic embryo	Grand Naine 30th Day somatic embryos	Grand Naine 45th Day somatic embryos	Grand Naine 60th Day somatic embryos
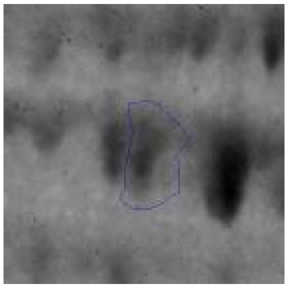	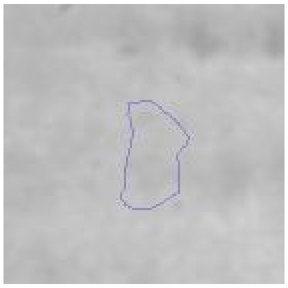	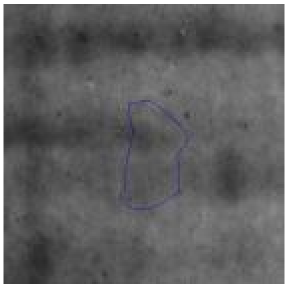	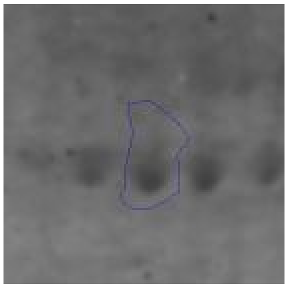

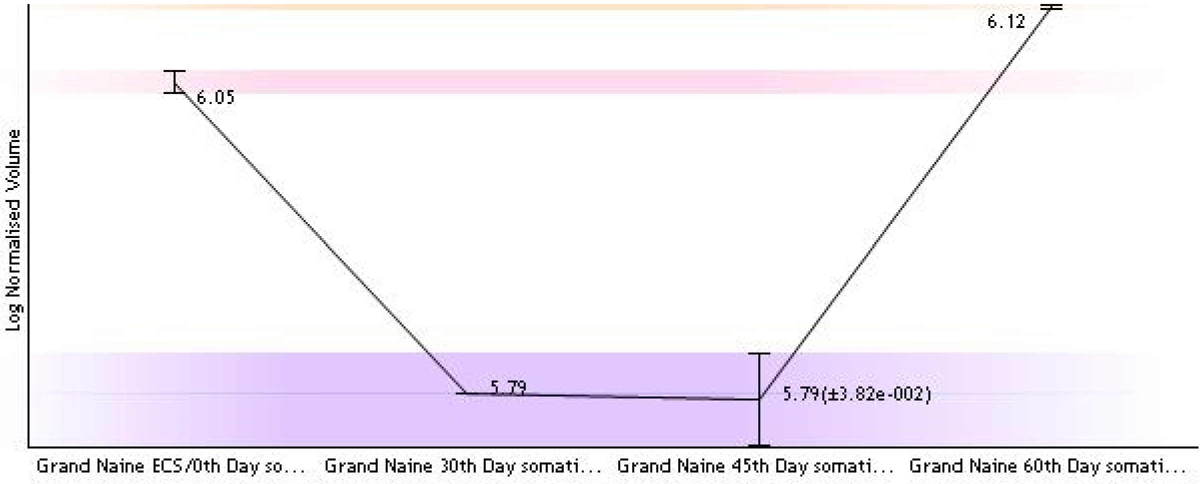

Identifier 394

Position (1086, 254)
Notes Spot 21

Grand Naine ECS/0th Day somatic embryo	Grand Naine 30th Day somatic embryos	Grand Naine 45th Day somatic embryos	Grand Naine 60th Day somatic embryos
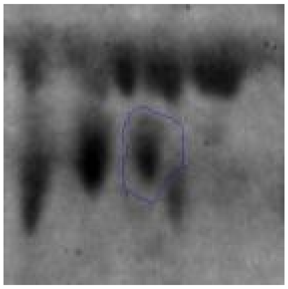	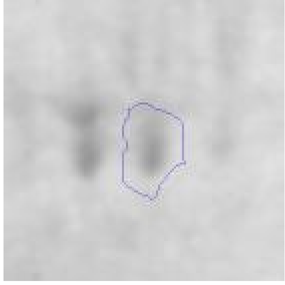	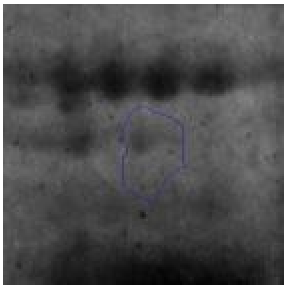	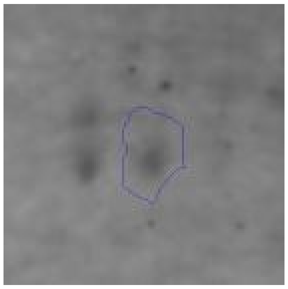

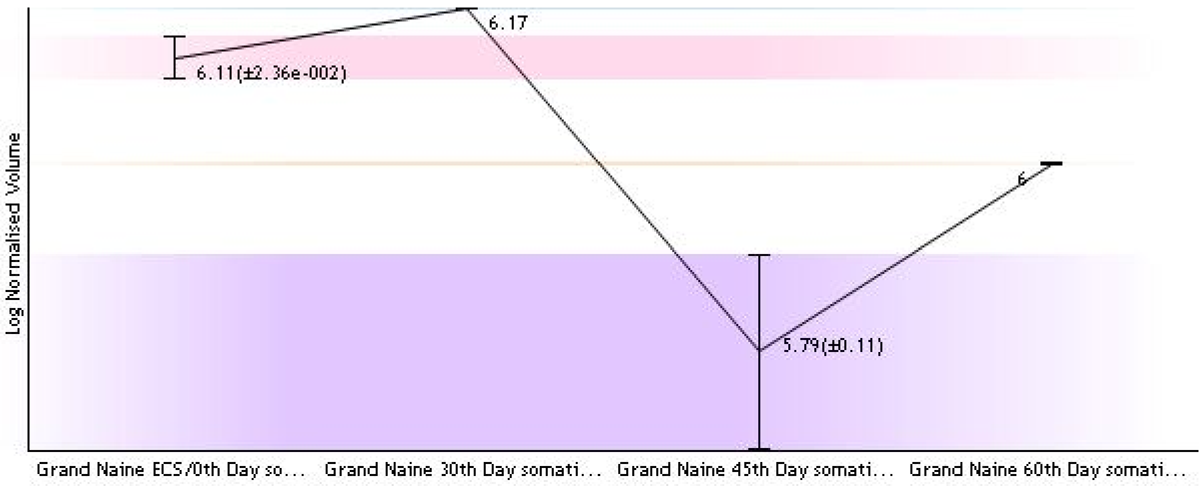

Identifier 1432

Position (1387, 860)
Notes Spot 20

Grand Naine ECS/0th Day somatic embryo	Grand Naine 30th Day somatic embryos	Grand Naine 45th Day somatic embryos	Grand Naine 60th Day somatic embryos

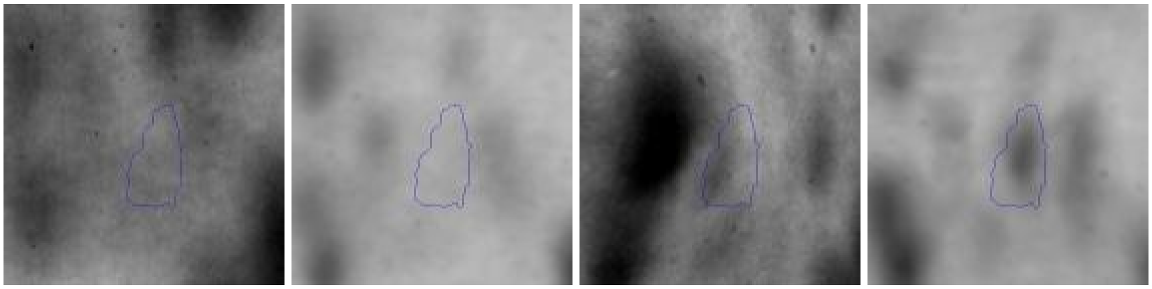

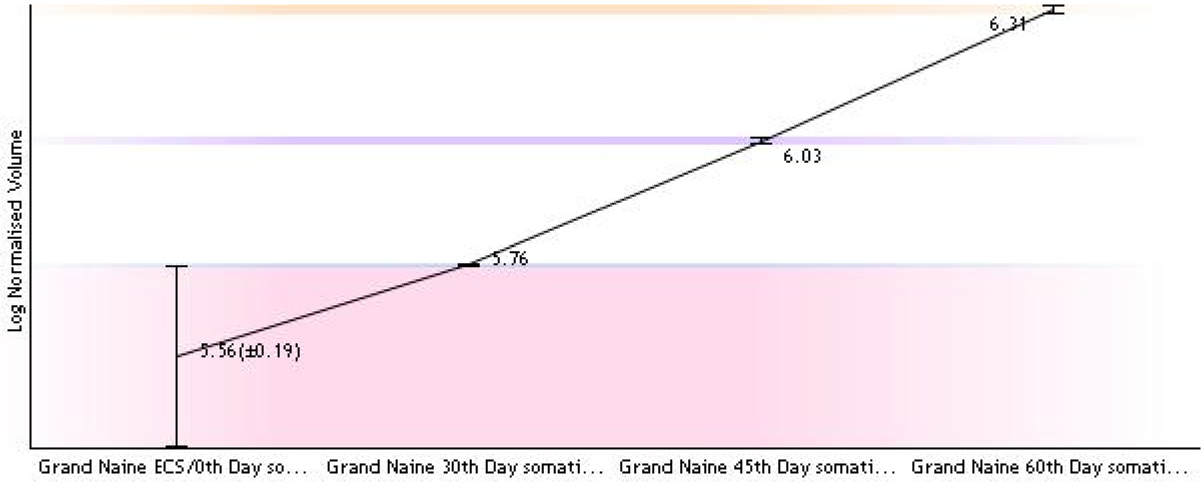

Identifier 1876

Position (560, 1153)
Notes Spot 23

Grand Naine ECS/0th Day somatic embryo	Grand Naine 30th Day somatic embryos	Grand Naine 45th Day somatic embryos	Grand Naine 60th Day somatic embryos

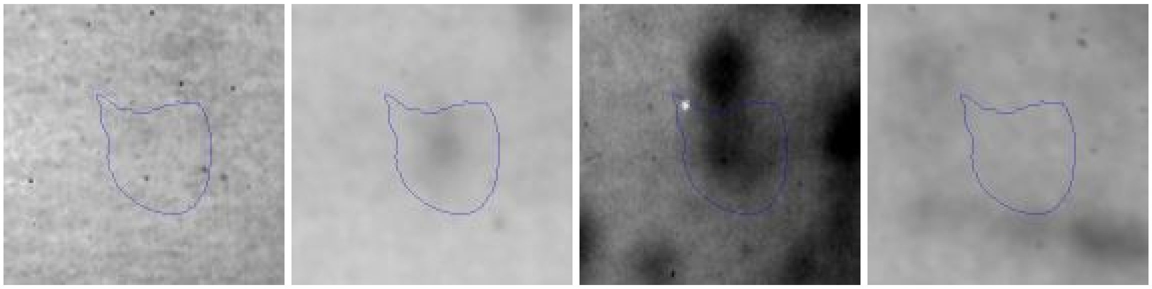

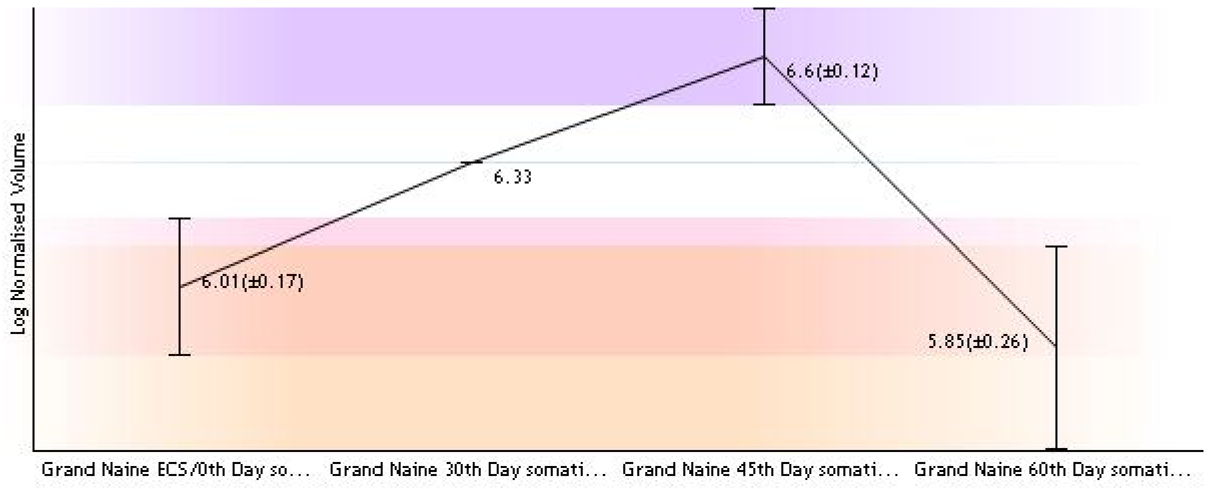

Identifier 2015

Position (1792, 1245)
Notes Spot 4

Grand Naine ECS/0th Day somatic embryo	Grand Naine 30th Day somatic embryos	Grand Naine 45th Day somatic embryos	Grand Naine 60th Day somatic embryos

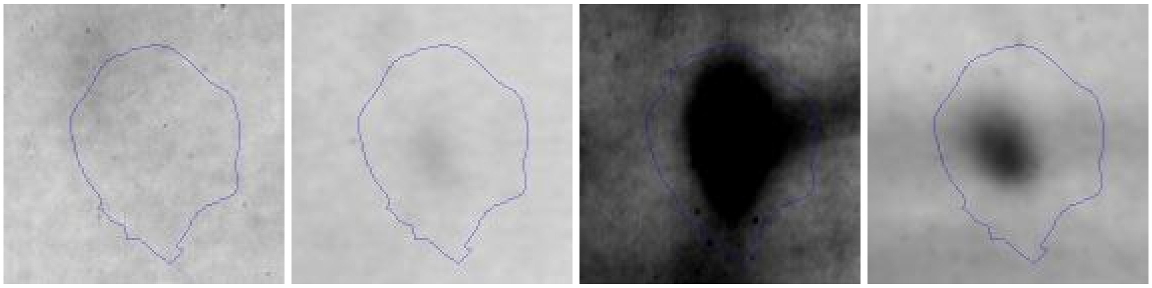

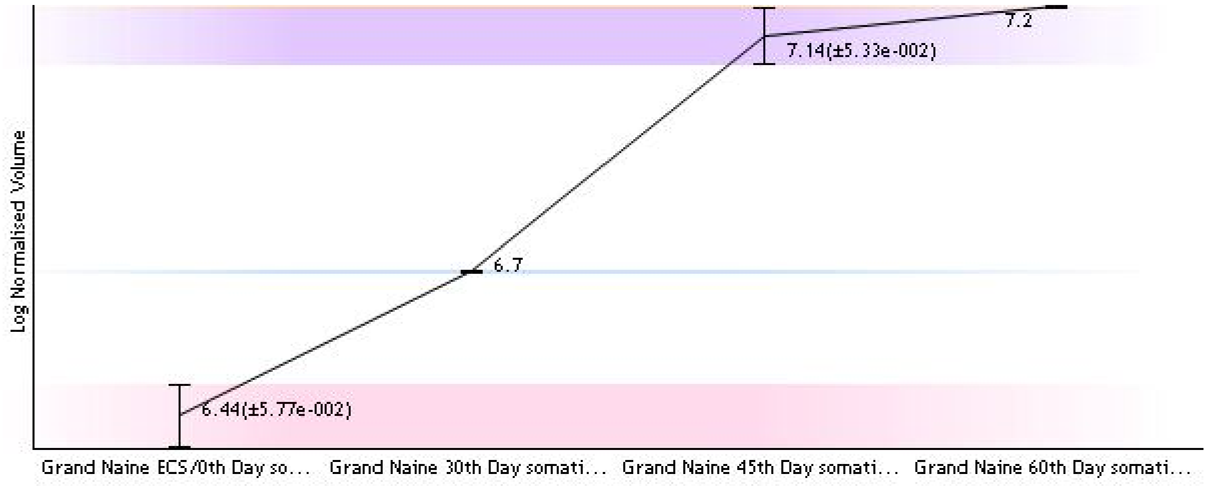

Identifier 2056

Position (1391, 1266)

Notes Spot 18

Grand Naine ECS/0th Day somatic embryo	Grand Naine 30th Day somatic embryos	Grand Naine 45th Day somatic embryos	Grand Naine 60th Day somatic embryos
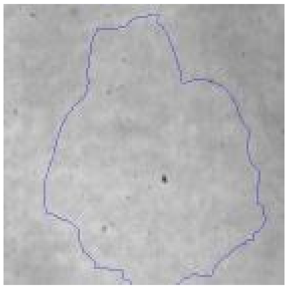	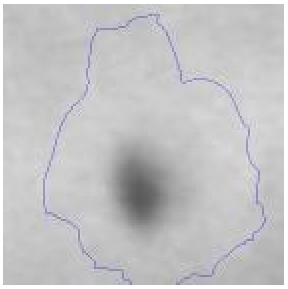	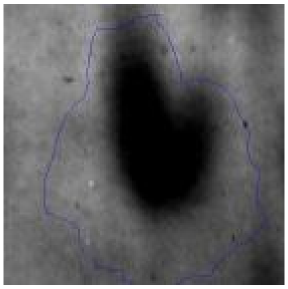	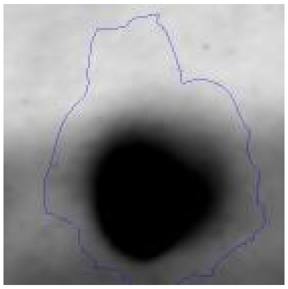

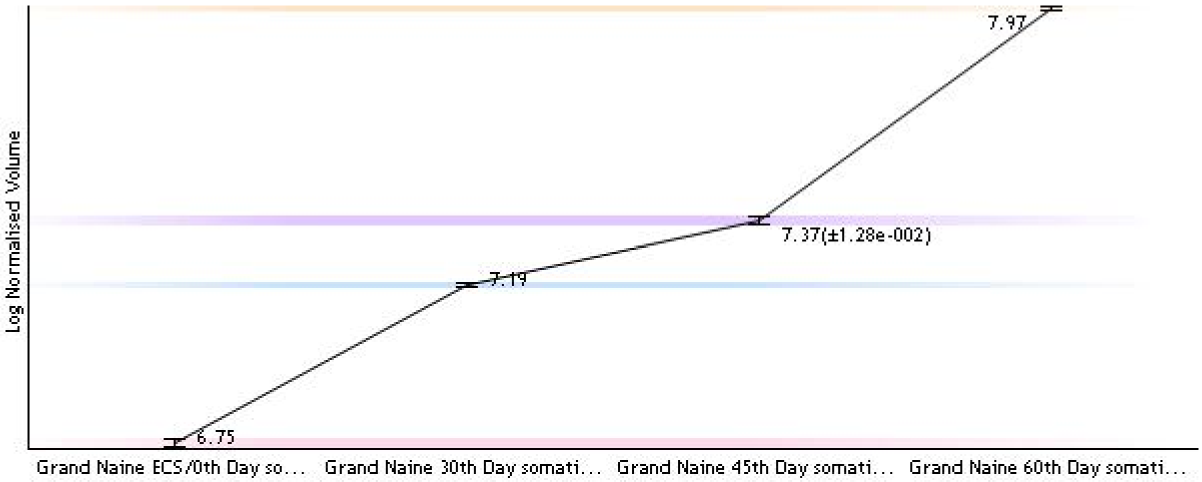

Identifier 2060

Position (1294, 1269)

Notes Spot 19

Grand Naine ECS/0th Day somatic embryo	Grand Naine 30th Day somatic embryos	Grand Naine 45th Day somatic embryos	Grand Naine 60th Day somatic embryos

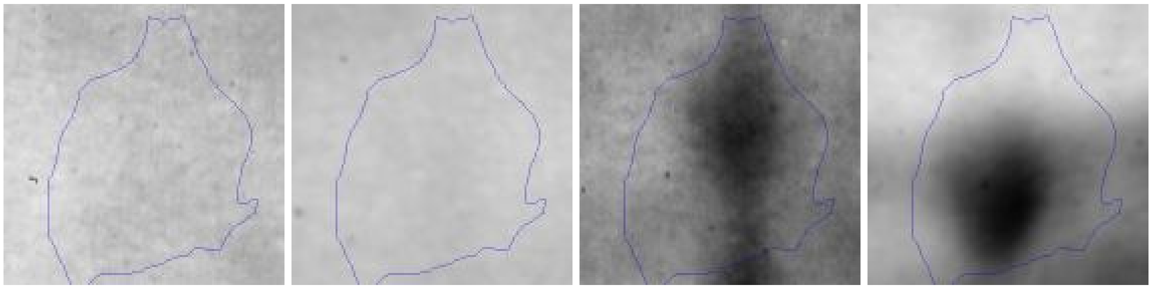

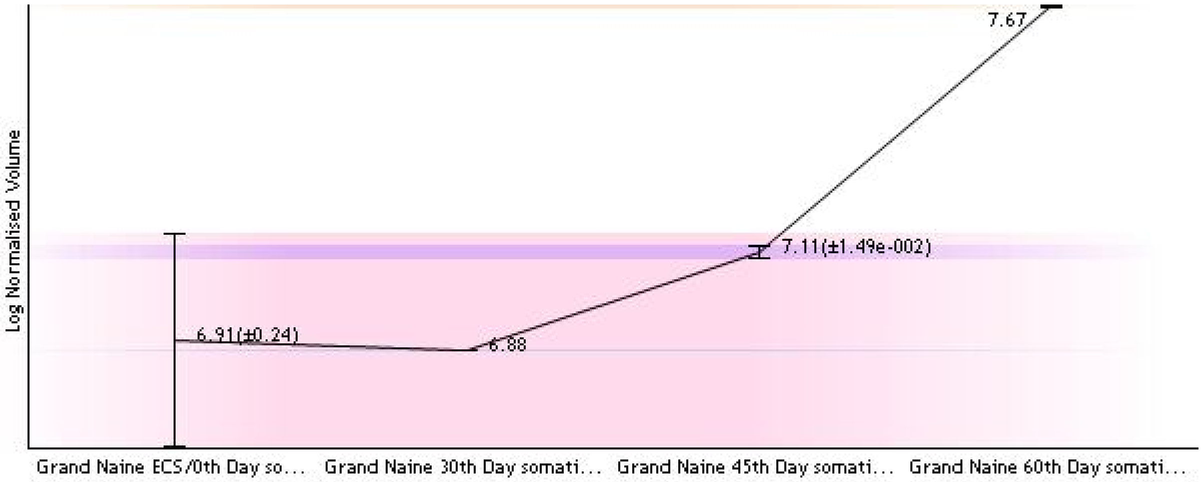

Identifier 2095

Position (665, 1296)
Notes Spot 9

Grand Naine ECS/0th Day somatic embryo	Grand Naine 30th Day somatic embryos	Grand Naine 45th Day somatic embryos	Grand Naine 60th Day somatic embryos

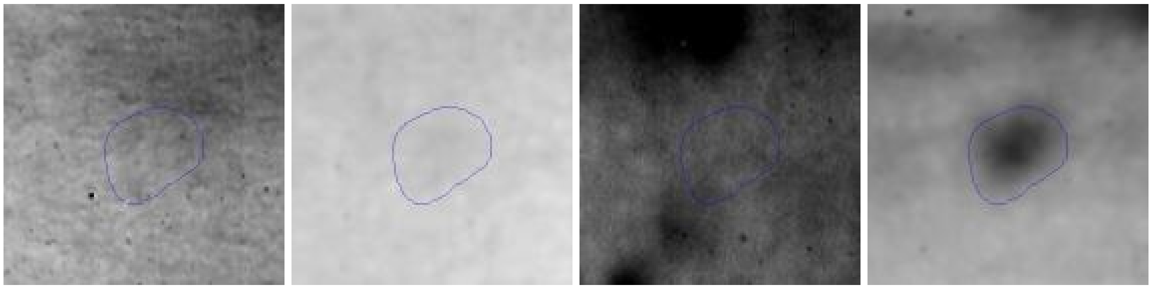

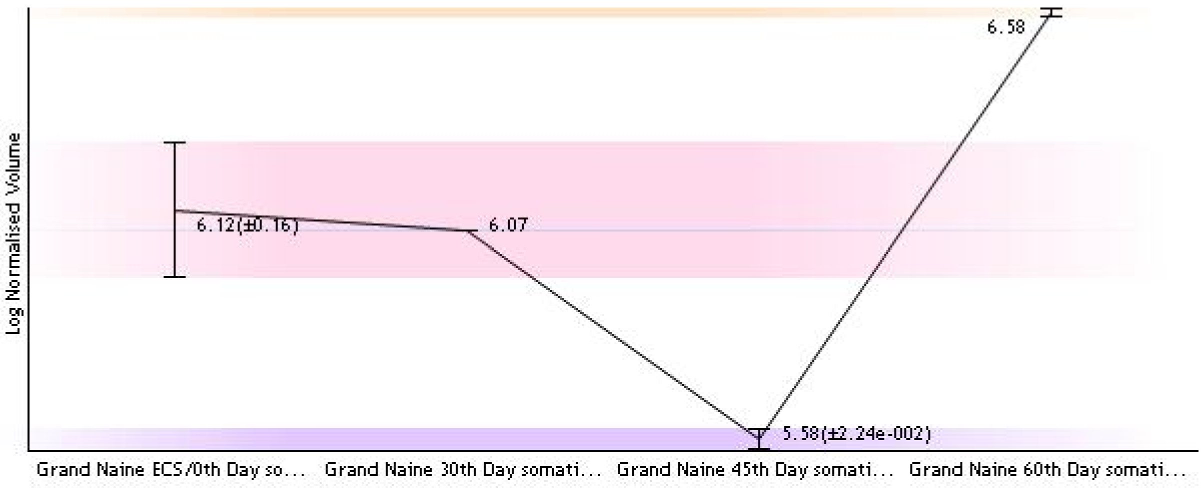

Identifier 2282

Position (1374, 1428)
Notes Spot 25

Grand Naine ECS/0th Day somatic embryo	Grand Naine 30th Day somatic embryos	Grand Naine 45th Day somatic embryos	Grand Naine 60th Day somatic embryos

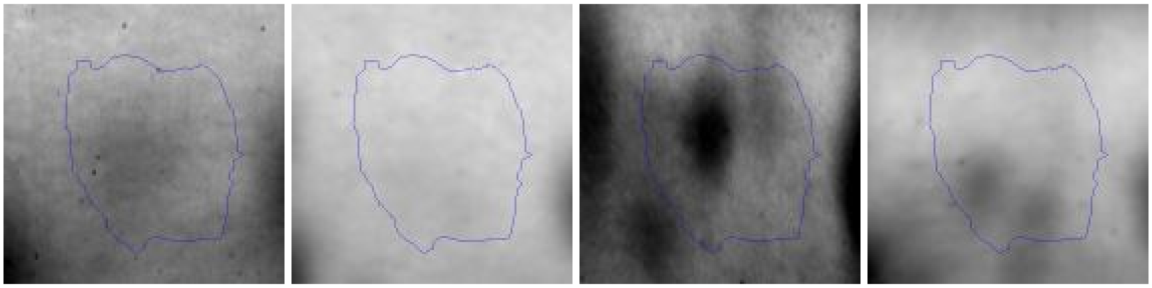

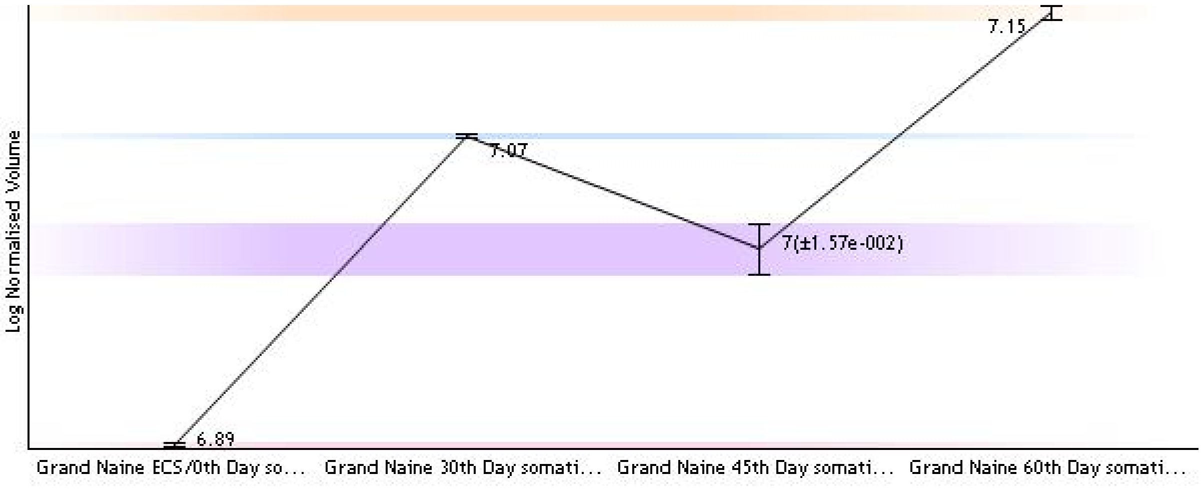

Identifier 3158

Position (2031, 1874)

Notes Spot 6

Grand Naine ECS/0th Day somatic embryo	Grand Naine 30th Day somatic embryos	Grand Naine 45th Day somatic embryos	Grand Naine 60th Day somatic embryos
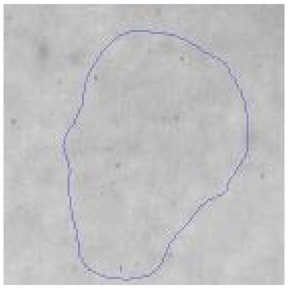	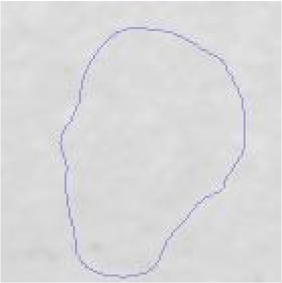	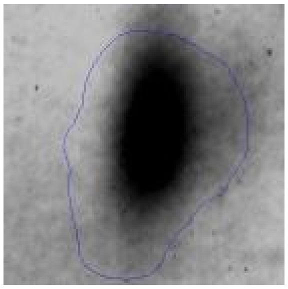	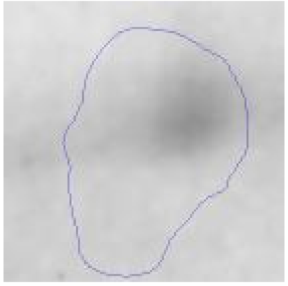

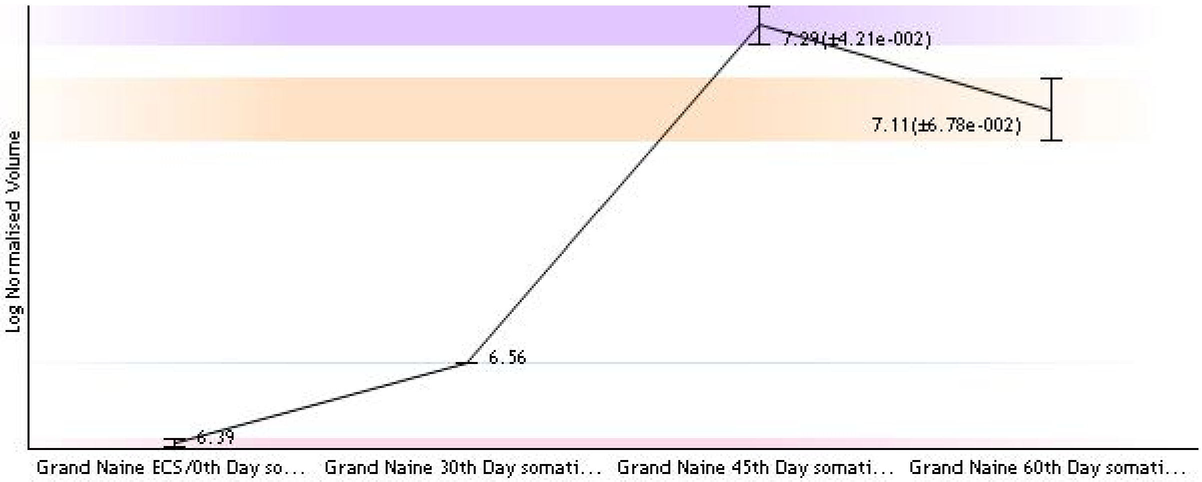

Identifier 3248

Position (1340, 1922)

Notes Spot 3

Grand Naine ECS/0th Day somatic embryo	Grand Naine 30th Day somatic embryos	Grand Naine 45th Day somatic embryos	Grand Naine 60th Day somatic embryos

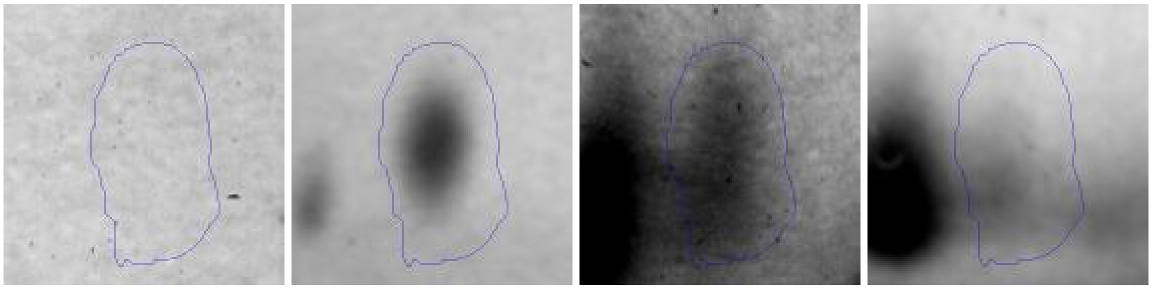

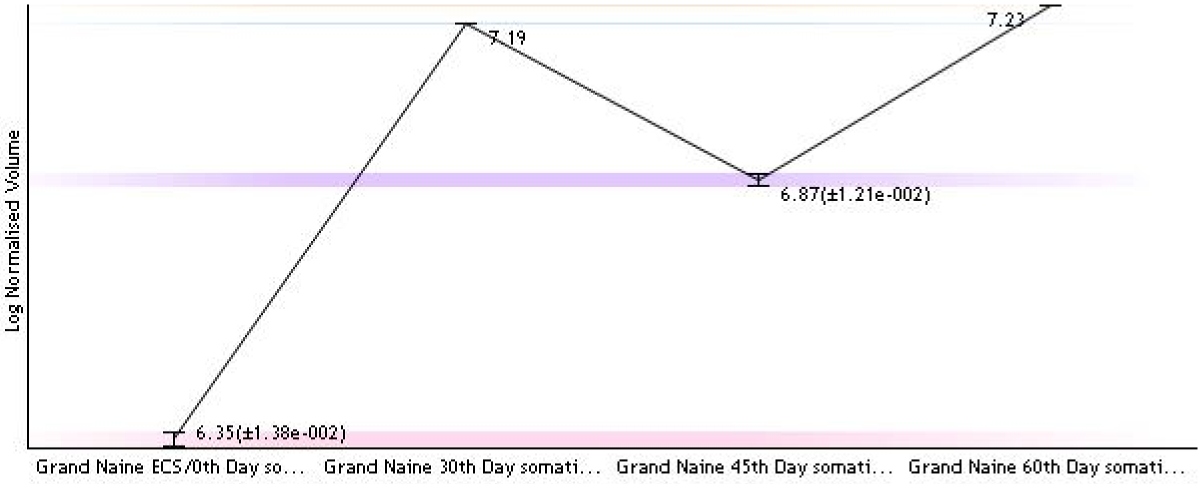

Identifier 3327

Position (1675, 1972)
Notes Spot 1

Grand Naine ECS/0th Day somatic embryo	Grand Naine 30th Day somatic embryos	Grand Naine 45th Day somatic embryos	Grand Naine 60th Day somatic embryos

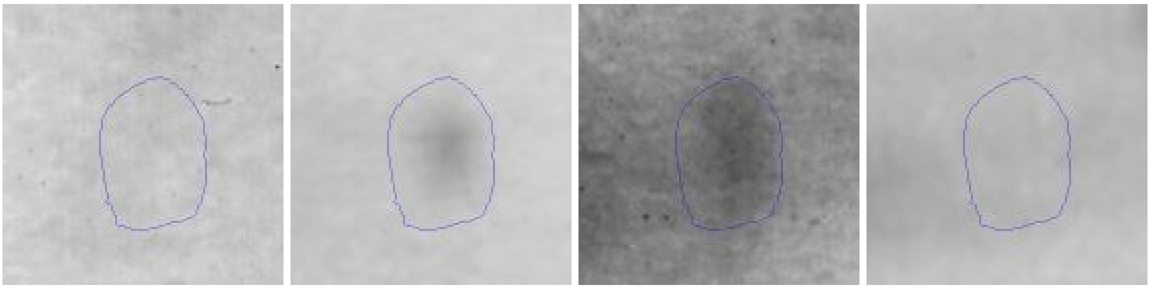

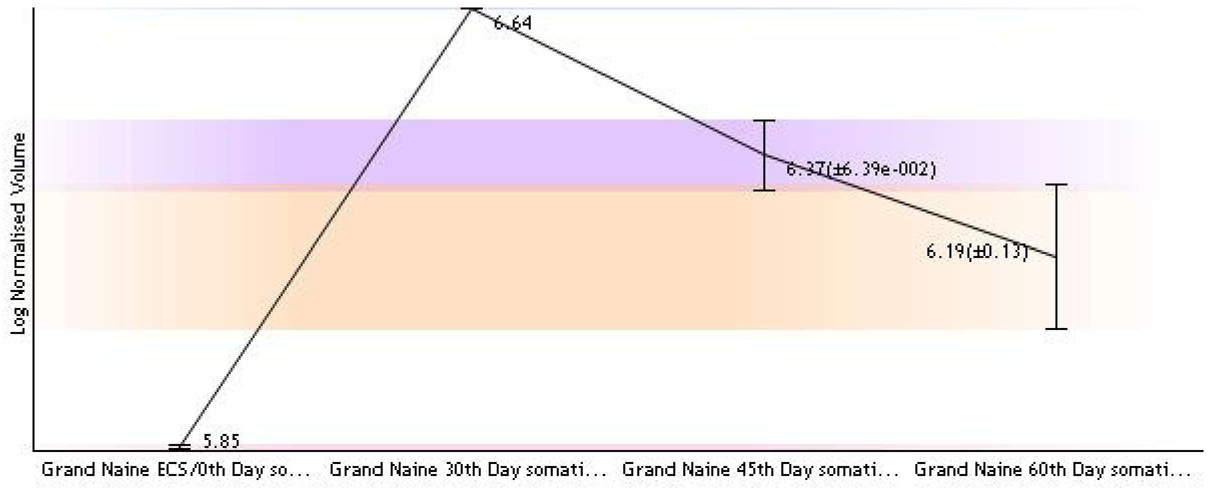

Identifier 3865

Position (1455, 1952)
Notes Spot 2

Grand Naine ECS/0th Day somatic embryo	Grand Naine 30th Day somatic embryos	Grand Naine 45th Day somatic embryos	Grand Naine 60th Day somatic embryos

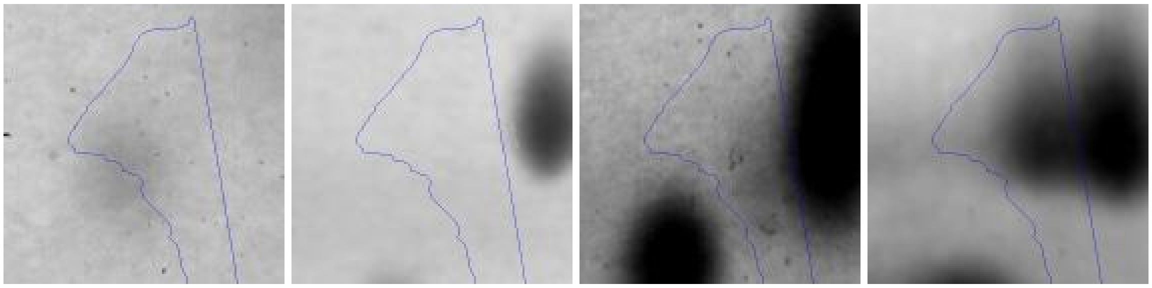

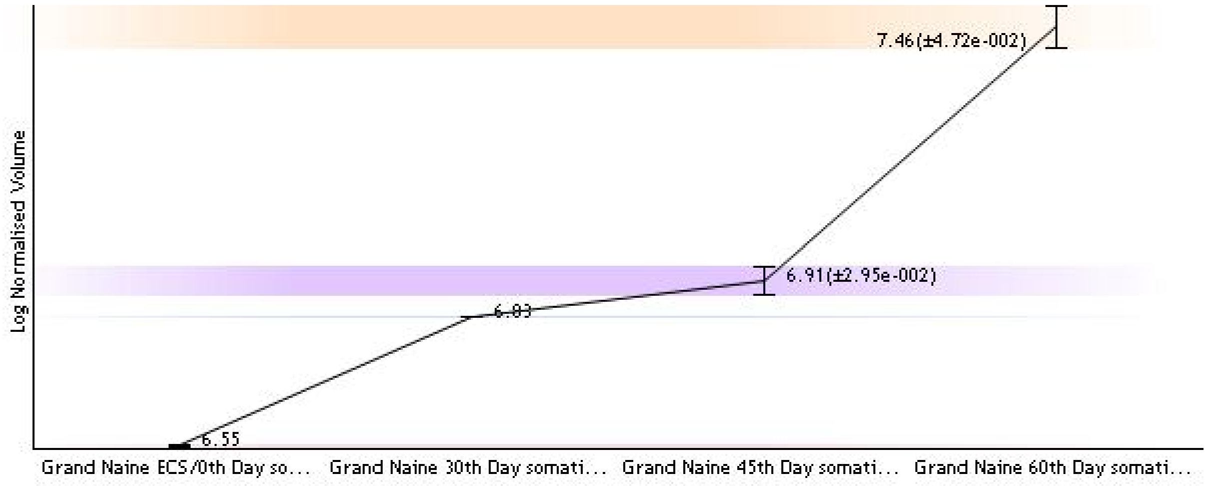

Identifier 3874

Position (650, 1191)

Notes Spot 5

Grand Naine ECS/0th Day somatic embryo	Grand Naine 30th Day somatic embryos	Grand Naine 45th Day somatic embryos	Grand Naine 60th Day somatic embryos
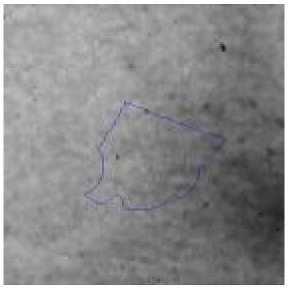	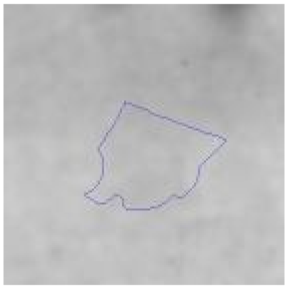	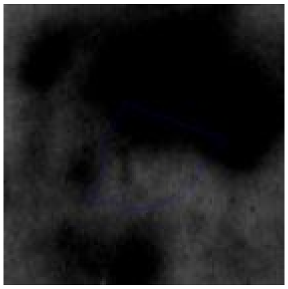	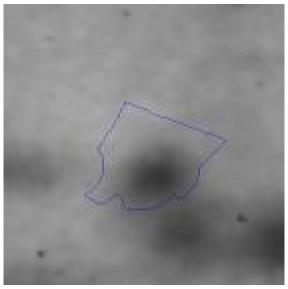

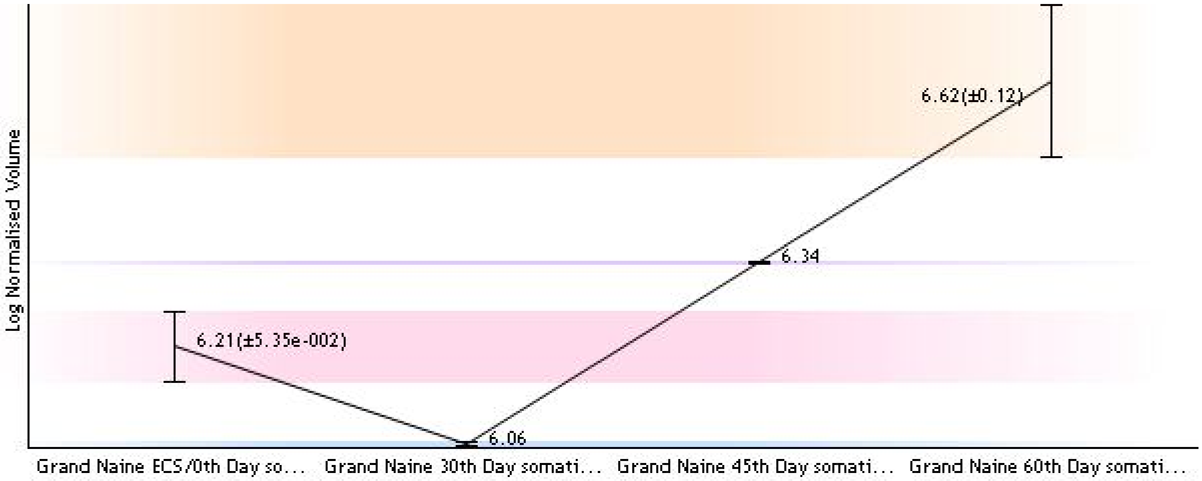

Identifier 3879

Position (1719, 963)

Notes Spot 7

Grand Naine ECS/0th Day somatic embryo	Grand Naine 30th Day somatic embryos	Grand Naine 45th Day somatic embryos	Grand Naine 60th Day somatic embryos

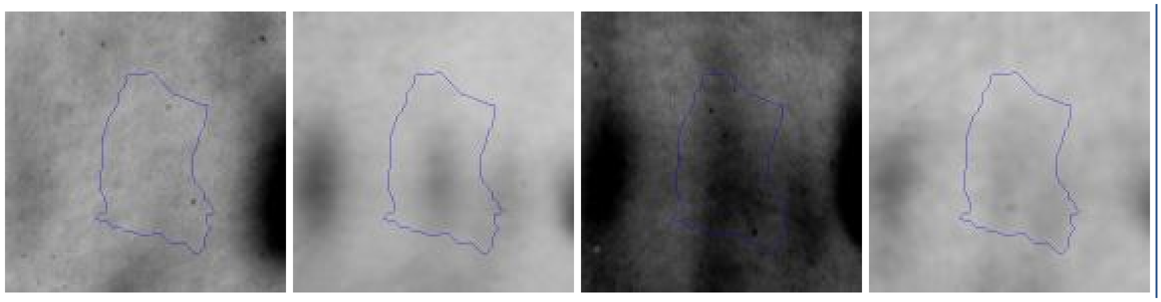

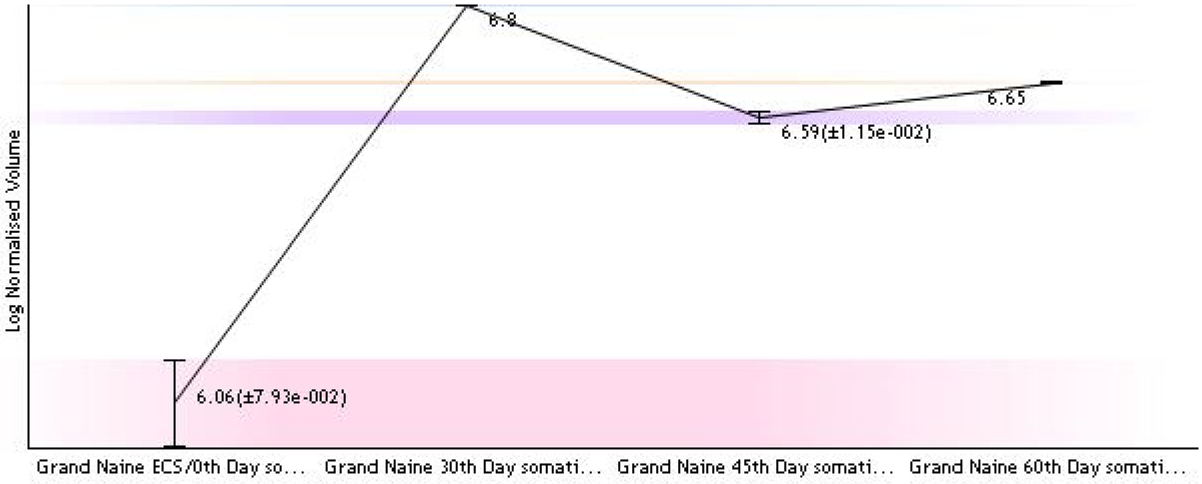

Identifier 3881

Position (1503, 897)
Notes Spot 8

Grand Naine ECS/0th Day somatic embryo	Grand Naine 30th Day somatic embryos	Grand Naine 45th Day somatic embryos	Grand Naine 60th Day somatic embryos

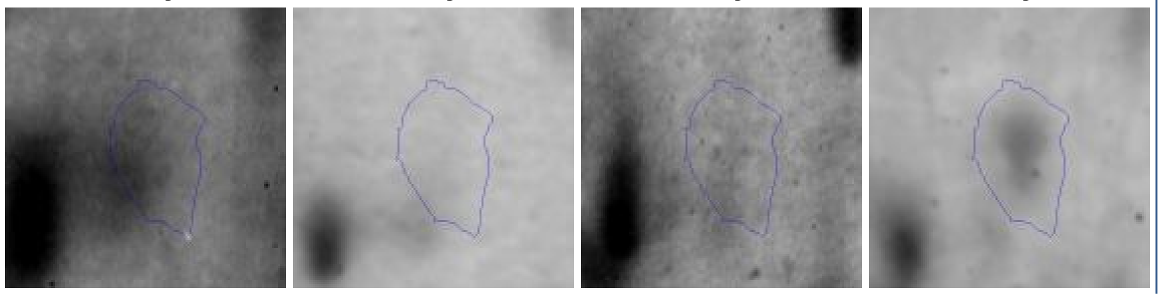

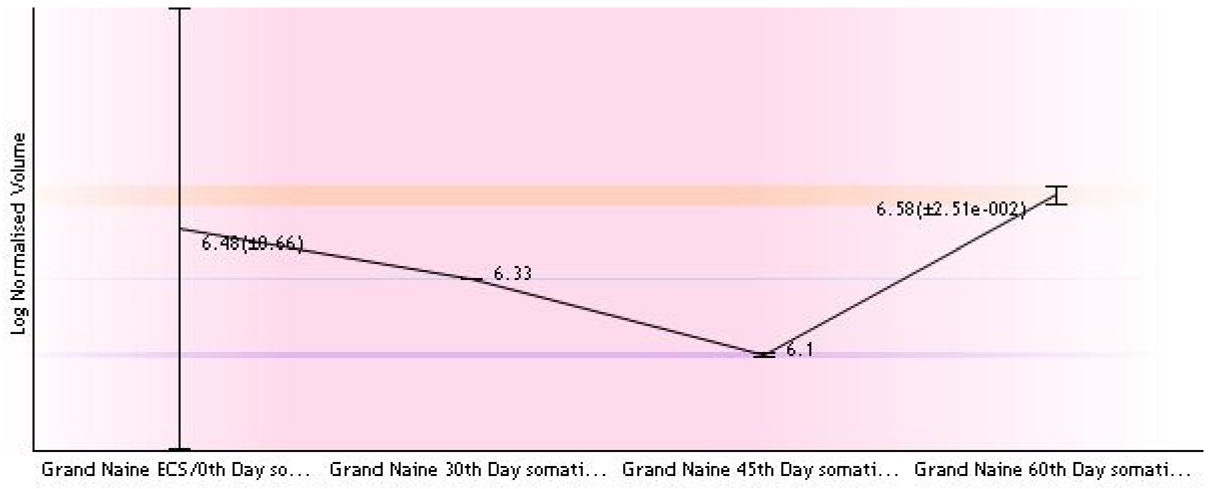

Identifier 3888

Position (717, 1218)
Notes Spot 10

Grand Naine ECS/0th Day somatic embryo	Grand Naine 30th Day somatic embryos	Grand Naine 45th Day somatic embryos	Grand Naine 60th Day somatic embryos

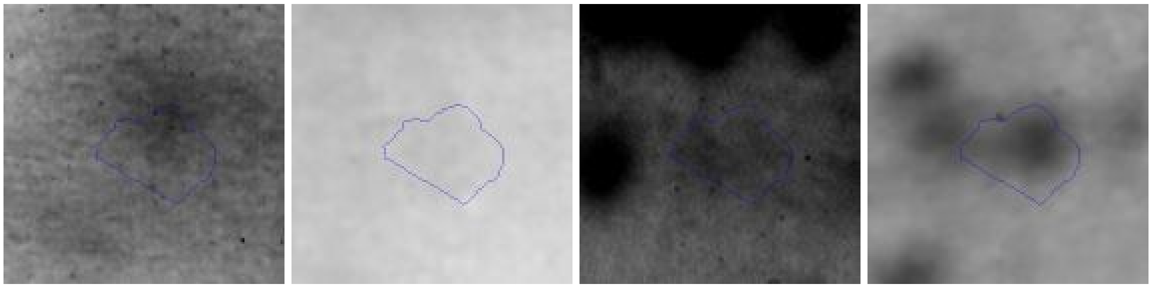

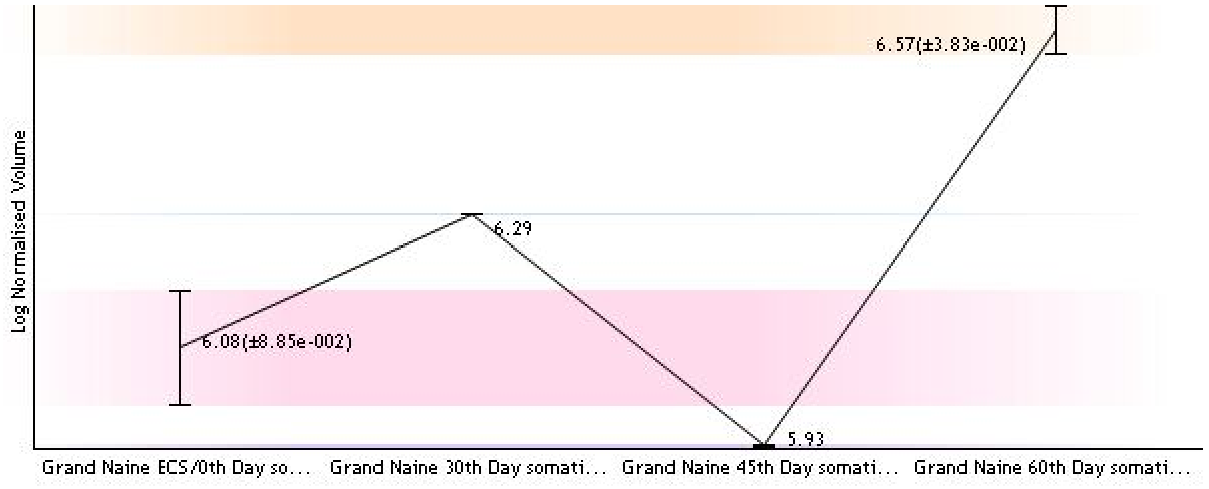

Identifier 3896

Position (685, 1223)
Notes Spot 11

Grand Naine ECS/0th Day somatic embryo	Grand Naine 30th Day somatic embryos	Grand Naine 45th Day somatic embryos	Grand Naine 60th Day somatic embryos

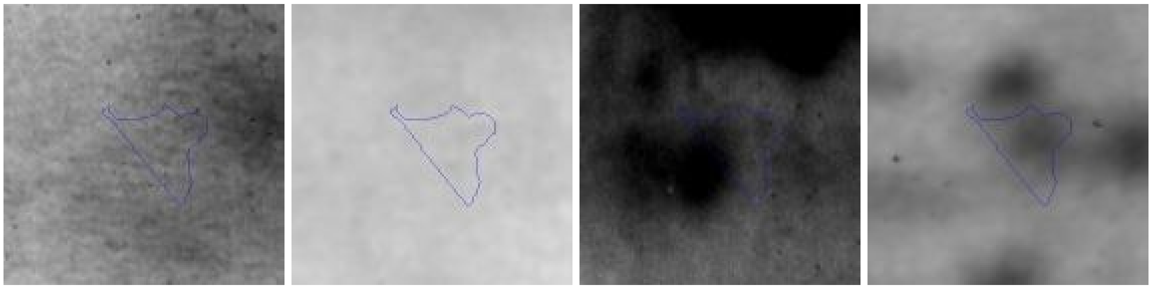

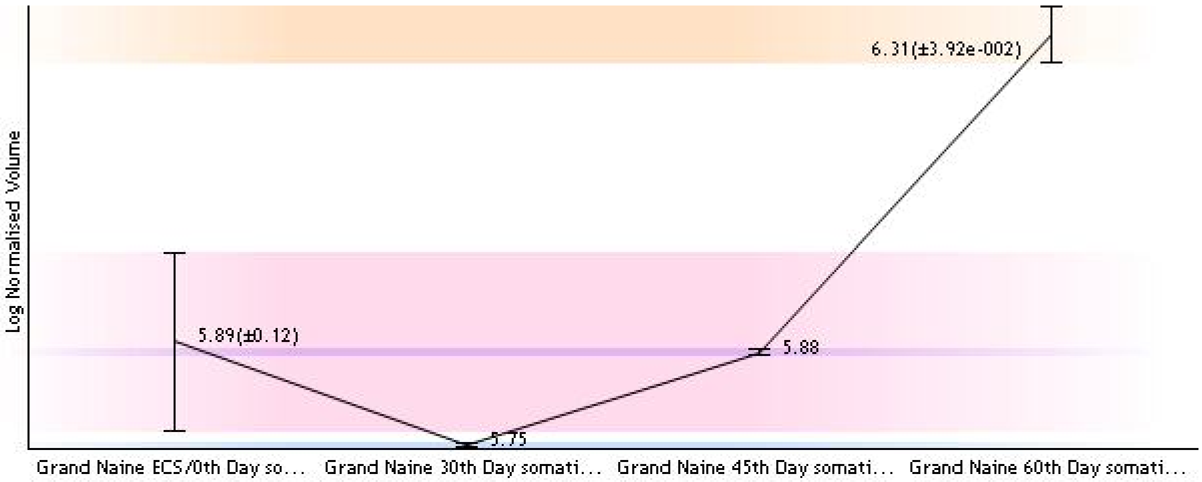

Identifier 3899

Position (846, 625)
Notes Spot 12

Grand Naine ECS/0th Day somatic embryo	Grand Naine 30th Day somatic embryos	Grand Naine 45th Day somatic embryos	Grand Naine 60th Day somatic embryos

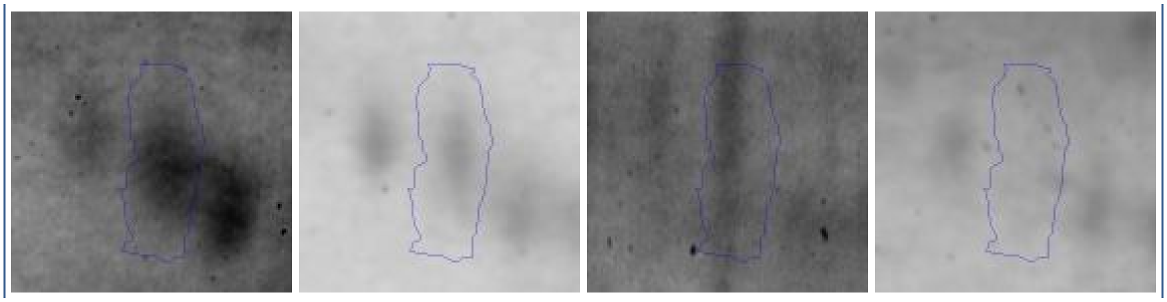

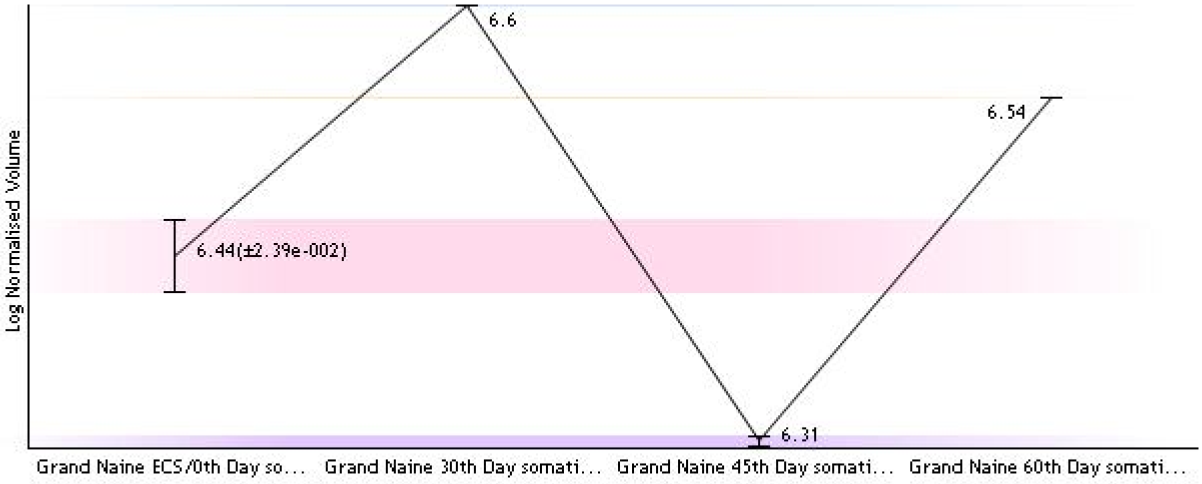

Identifier 3902

Position (981, 667)
Notes Spot 13

Grand Naine ECS/0th Day somatic embryo	Grand Naine 30th Day somatic embryos	Grand Naine 45th Day somatic embryos	Grand Naine 60th Day somatic embryos

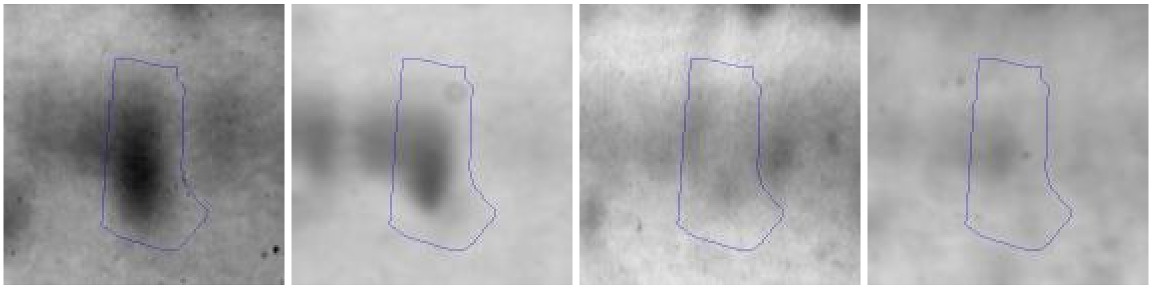

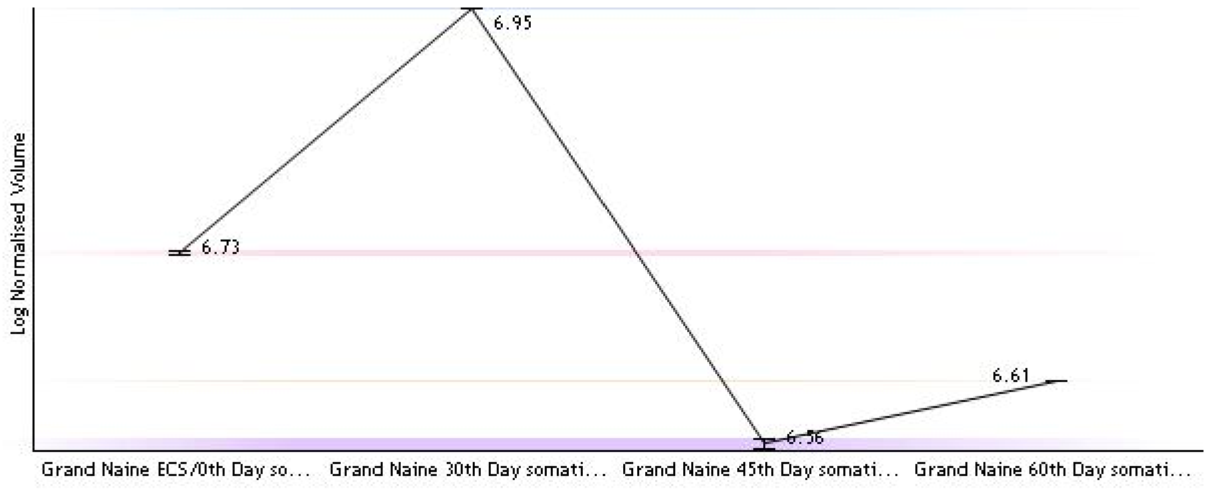

Identifier 3903

Position (1176, 1255)
Notes Spot 14

Grand Naine ECS/0th Day somatic embryo	Grand Naine 30th Day somatic embryos	Grand Naine 45th Day somatic embryos	Grand Naine 60th Day somatic embryos

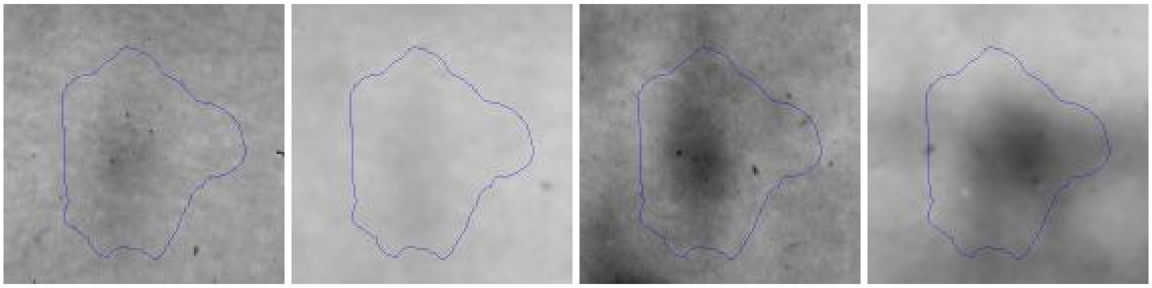

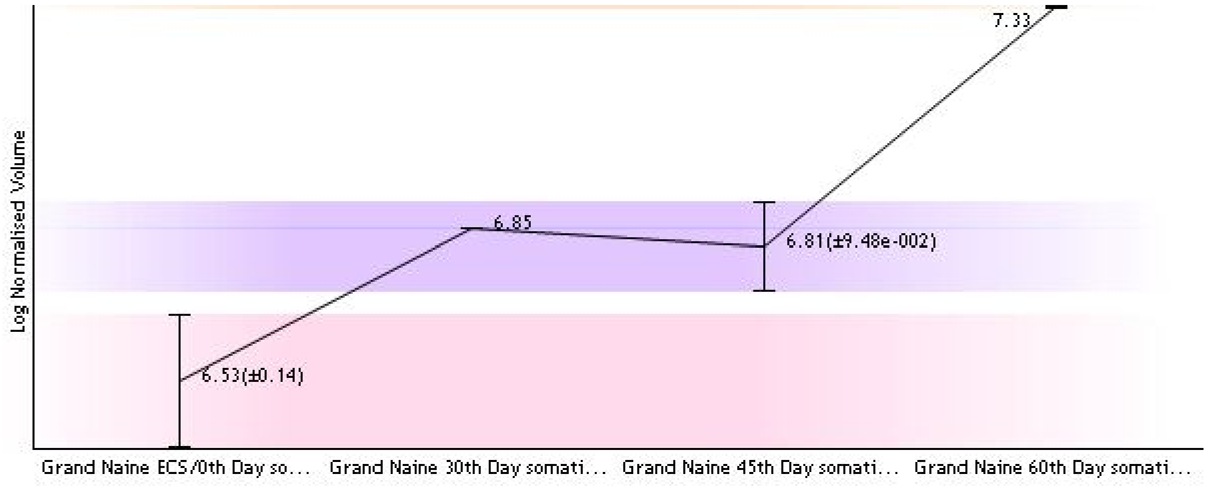

Identifier 3904

Position (1056, 1276)

Notes Spot 15

Grand Naine ECS/0th Day somatic embryo	Grand Naine 30th Day somatic embryos	Grand Naine 45th Day somatic embryos	Grand Naine 60th Day somatic embryos
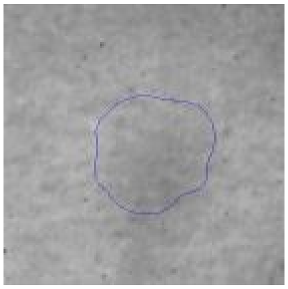	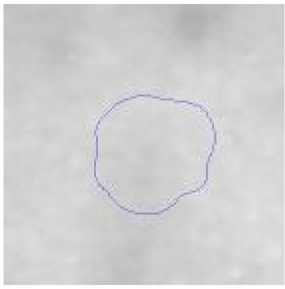	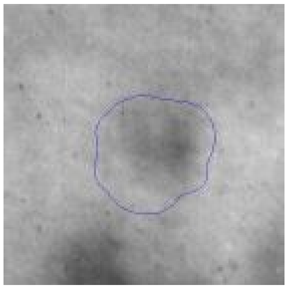	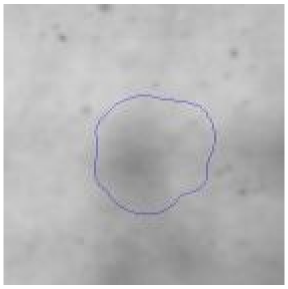

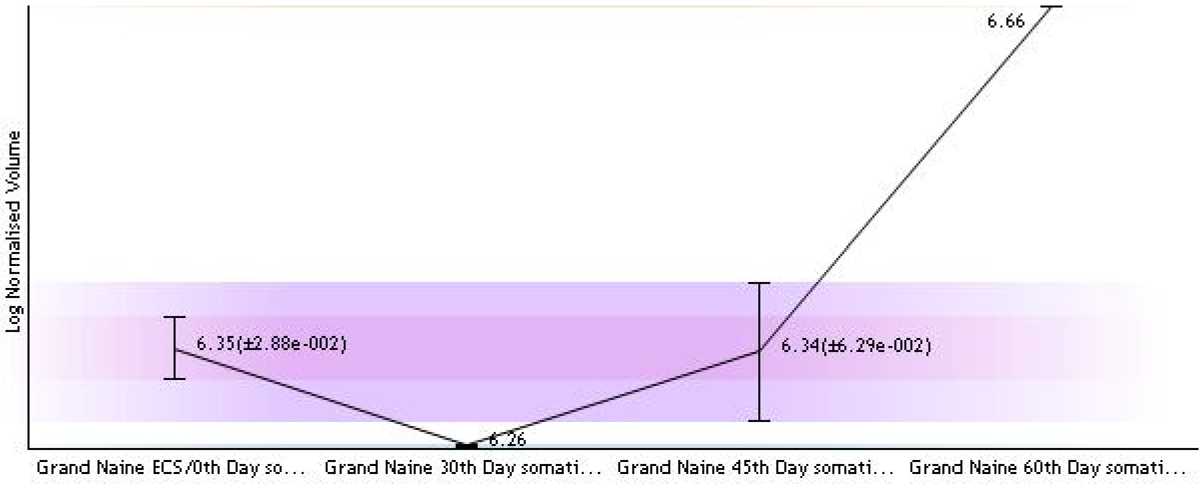

Identifier 3905

Position (1150, 1050)

Notes Spot 16

Grand Naine ECS/0th Day somatic embryo	Grand Naine 30th Day somatic embryos	Grand Naine 45th Day somatic embryos	Grand Naine 60th Day somatic embryos

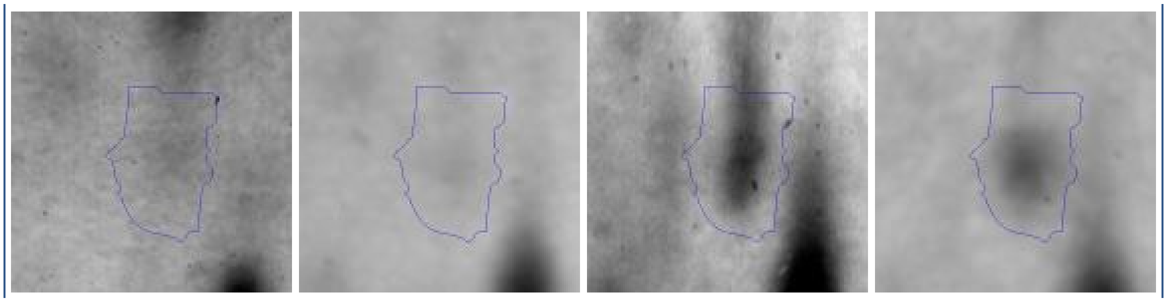

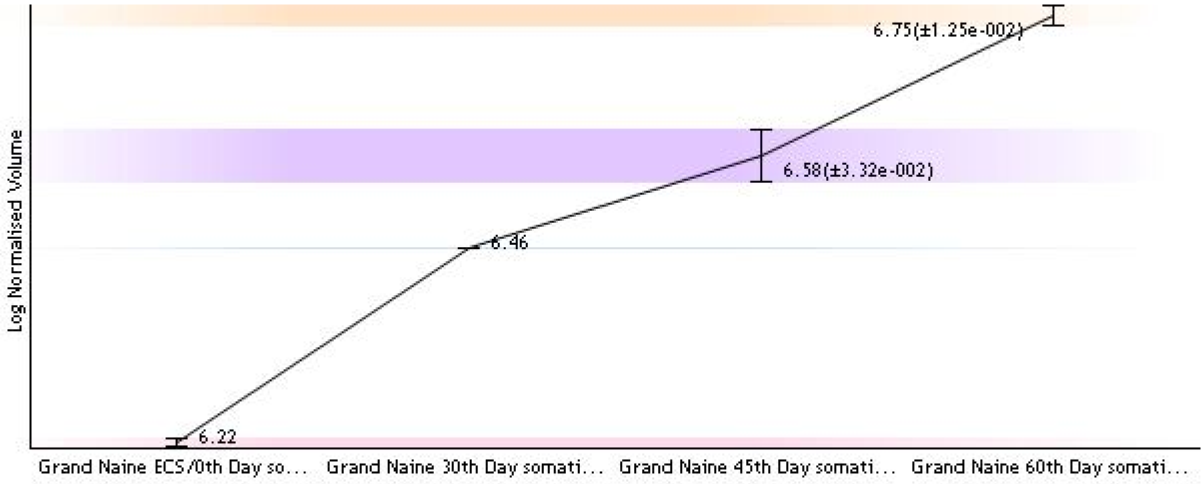

Identifier 3969

Position (1192, 1886)
Notes Spot 22

Grand Naine ECS/0th Day somatic embryo	Grand Naine 30th Day somatic embryos	Grand Naine 45th Day somatic embryos	Grand Naine 60th Day somatic embryos

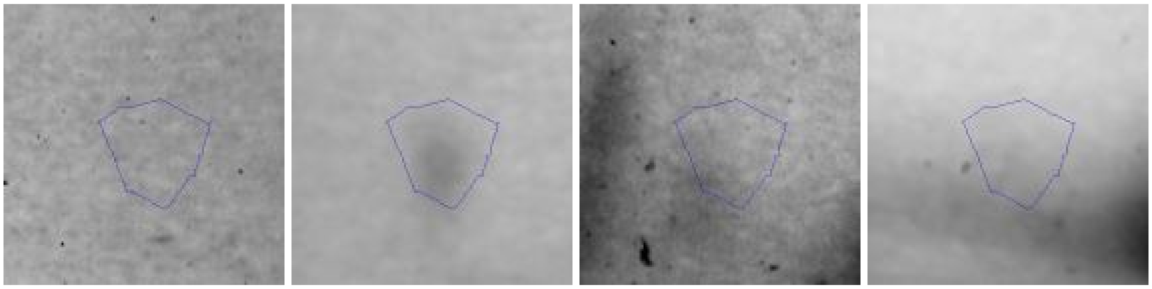

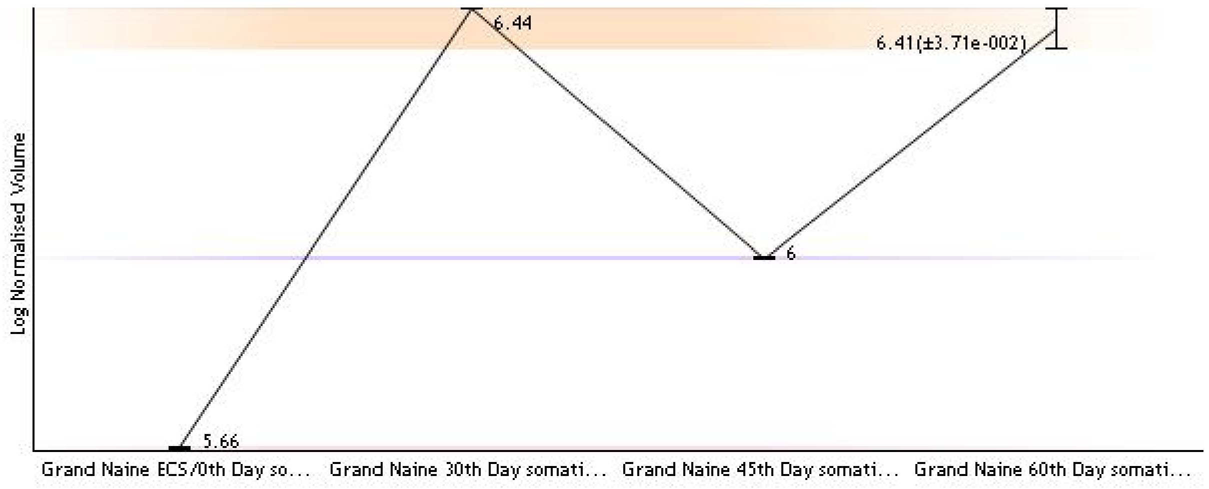

Identifier 3970

Position (1080, 1162)
Notes Spot 24

Grand Naine ECS/0th Day somatic embryo	Grand Naine 30th Day somatic embryos	Grand Naine 45th Day somatic embryos	Grand Naine 60th Day somatic embryos
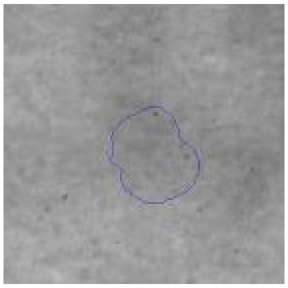	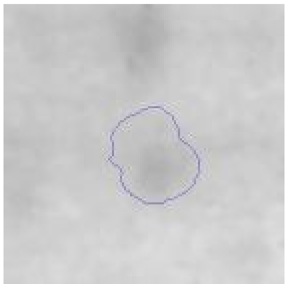	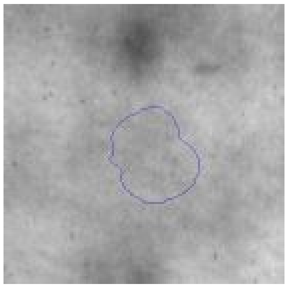	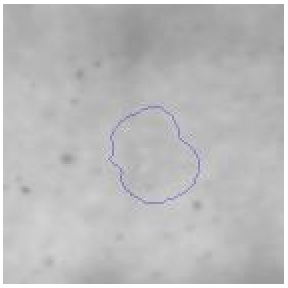

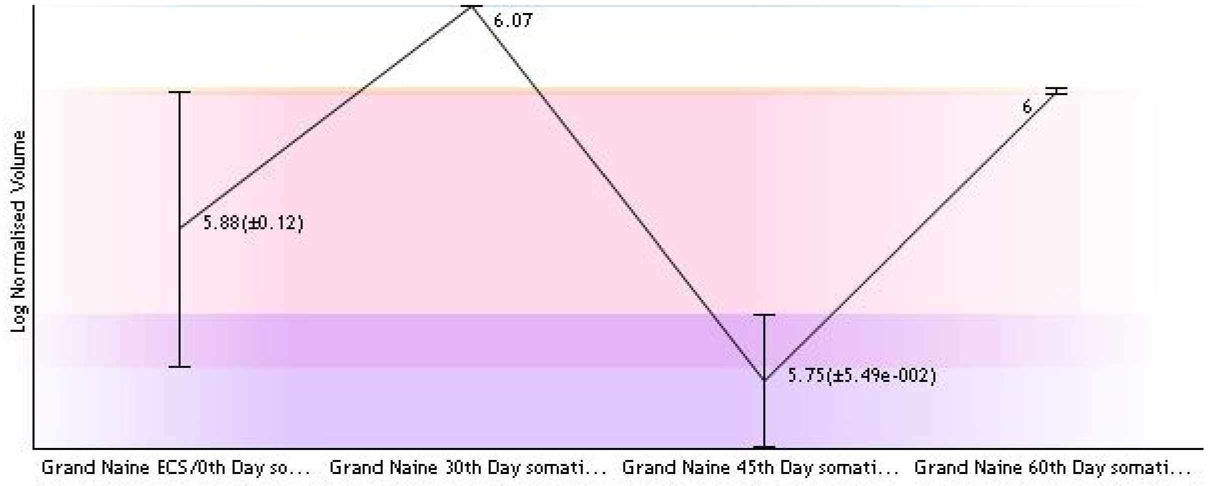

Supplement: Supplementary file 2 — Dataset 2. [file 41598_2020_61005_MOESM2_ESM.pdf]
